# Genomic Characterization of *Lactobacillus delbrueckii* TUA4408L and Evaluation of the Antiviral Activities of its Extracellular Polysaccharides in Porcine Intestinal Epithelial Cells

**DOI:** 10.3389/fimmu.2018.02178

**Published:** 2018-09-24

**Authors:** Paulraj Kanmani, Leonardo Albarracin, Hisakazu Kobayashi, Elvira Maria Hebert, Lucila Saavedra, Ryoya Komatsu, Brian Gatica, Ayako Miyazaki, Wakako Ikeda-Ohtsubo, Yoshihito Suda, Hisashi Aso, Shintaro Egusa, Takashi Mishima, Alexis Salas-Burgos, Hideki Takahashi, Julio Villena, Haruki Kitazawa

**Affiliations:** ^1^Food and Feed Immunology Group, Laboratory of Animal Products Chemistry, Graduate School of Agricultural Science, Tohoku University, Sendai, Japan; ^2^Livestock Immunology Unit, International Education and Research Center for Food Agricultural Immunology (CFAI), Graduate School of Agricultural Science, Tohoku University, Sendai, Japan; ^3^Reference Centre for Lactobacilli (CERELA-CONICET), Tucuman, Argentina; ^4^Scientific Computing Laboratory, Computer Science Department, Faculty of Exact Sciences and Technology, National University of Tucuman, Tucuman, Argentina; ^5^Department of Pharmacology, University of Concepcion, Concepcion, Chile; ^6^Viral Diseases and Epidemiology Research Division, National Institute of Animal Health, NARO, Tsukuba, Japan; ^7^Department of Food, Agriculture, and Environment, Miyagi University, Sendai, Japan; ^8^Cell Biology Laboratory, Graduate School of Agricultural Science, Tohoku University, Sendai, Japan; ^9^Research & Development Division, Marusan-Ai Co., Ltd., Okazaki, Japan; ^10^Graduate School of Regional Innovation Studies, Mie University, Tsu, Japan; ^11^Laboratory of Plant Pathology, Graduate School of Agricultural Science, Tohoku University, Sendai, Japan; ^12^Plant Immunology Unit, International Education and Research Center for Food Agricultural Immunology (CFAI), Graduate School of Agricultural Science, Tohoku University, Sendai, Japan

**Keywords:** porcine intestinal epithelial cells, immunobiotics, TLR3, Rotavirus, antiviral activity, genome sequence, *Lactobacillus delbrueckii* TUA4408L

## Abstract

In lactic acid bacteria, the synthesis of exopolysaccharides (EPS) has been associated with some favorable technological properties as well as health-promoting benefits. Research works have shown the potential of EPS produced by lactobacilli to differentially modulate immune responses. However, most studies were performed in immune cells and few works have concentrated in the immunomodulatory activities of EPS in non-immune cells such as intestinal epithelial cells. In addition, the cellular and molecular mechanisms involved in the immunoregulatory effects of EPS have not been studied in detail. In this work, we have performed a genomic characterization of *Lactobacillus delbrueckii* subsp. *delbrueckii* TUA4408L and evaluated the immunomodulatory and antiviral properties of its acidic (APS) and neutral (NPS) EPS in porcine intestinal epithelial (PIE) cells. Whole genome sequencing allowed the analysis of the general features of *L. delbrueckii* TUA4408L genome as well as the characterization of its EPS genes. A typical EPS gene cluster was found in the TUA4408L genome consisting in five highly conserved genes *epsA*-*E*, and a variable region, which includes the genes for the polymerase *wzy*, the flippase *wzx*, and seven glycosyltransferases. In addition, we demonstrated here for the first time that *L. delbrueckii* TUA4408L and its EPS are able to improve the resistance of PIE cells against rotavirus infection by reducing viral replication and regulating inflammatory response. Moreover, studies in PIE cells demonstrated that the TUA4408L strain and its EPS differentially modulate the antiviral innate immune response triggered by the activation of Toll-like receptor 3 (TLR3). *L. delbrueckii* TUA4408L and its EPS are capable of increasing the activation of interferon regulatory factor (IRF)-3 and nuclear factor κB (NF-κB) signaling pathways leading to an improved expression of the antiviral factors interferon (IFN)-β, Myxovirus resistance gene A (MxA) and RNaseL.

## Introduction

Polysaccharides are widespread in nature, and their production has been described in several species of pathogenic and commensal bacteria. Variations in the sugar-building units, glycosidic linkage, anomeric configuration, monosaccharide decoration, and molecular weight result in an enormous diversity of polysaccharides. Because of this heterogeneity, bacterial polysaccharides display diverse chemical, physical and biological properties (1, 2). Bacteria are able to synthesize cytoplasmic storage polysaccharides and exocellular polysaccharides including the tightly linked capsular polysaccharides (CPS) and the loosely associated with the cell surface exopolysaccharides (EPS) (1, 2). It was established that exocellular polysaccharides are involved in the interaction of bacteria with their environment. CPS and EPS have been shown to participate in the formation of bacterial biofilms, adhesion to abiotic and biotic surfaces as well as in the interaction with the immune system (3–5).

In lactic acid bacteria (LAB), the synthesis of EPS has long been associated with some favorable technological properties, especially in food production where they act as viscosifying, stabilizing, emulsifying, or gelling agents. In addition, functional and health-promoting benefits have been attributed to the EPS produced by some LAB strains (2, 5). In this regard, it was reported that surface EPS produced by lactobacilli are able to modulate the immune system. Studies by Yasuda et al. (6) demonstrated that high molecular mass polysaccharides from *Lactobacillus casei* strain Shirota have anti-inflammatory effects while mutant types of this bacterium lacking EPS are potent inducers of interleukin (IL)-12, tumor necrosis factor (TNF)-α, and IL-6 in macrophages. It was also shown that EPS from *L. rhamnosus* RW-9595M exerted immunosuppressive properties in macrophages by inducing high levels of IL-10 (7). In addition to the anti-inflammatory activities, some research works have demonstrated that EPS from lactobacilli are also capable of stimulating the immune system. It was shown that EPS produced by *L. paracasei* DG is able to enhance the expression of TNF-α, IL-6, IL-8, and macrophage inflammatory protein 3α (MIP-3α) in the human monocytic cell line THP-1 (8). EPS derived from *L. rhamnosus* KL37 was also capable of improving TNF-α, IL-6, and IL-12 in macrophages in a mitogen-activated protein kinases (MAPK)-dependent manner (9).

Research works clearly show the potential of EPS produced by lactobacilli to favorably modulate the immune system. However, there are several points that have not yet been studied in depth in relation to the application of immunomodulatory EPS from lactobacilli: (a) most studies were performed in immune cells and few works have concentrated in the immunomodulatory activities of EPS in non-immune cells such as intestinal epithelial cells; (b) the cellular and molecular mechanisms involved in the immunoregulatory effects have not been studied in detail; (c) most research works evaluated the immunological effects of EPS produced by *L. casei* and *L. rhamnosus* while other species of *Lactobacillus* that are also capable of producing important quantities of EPS such as *L. delbrueckii* has not been investigated, and (d) very few studies have demonstrated that EPS from lactobacilli can exert a real beneficial effect through the modulation of the immune system such as increasing the resistance to bacterial or viral infections.

We have conducted research aimed at deepening in the knowledge of the points mentioned above. In previous studies, we evaluated the immunomodulatory properties of several lactobacilli strains according to their capacity to differentially modulate the immune response of porcine intestinal epithelial (PIE) cells triggered by the activation of Toll-like receptor 4 (TLR4). Our previous studies showed that the PIE cell line preserves all the immunological characteristics of primary epithelial cells and therefore, this cell line is a useful *in vitro* tool to evaluate immune responses (10). In addition, this cell line has been used by our group to evaluate immunomodulatory microorganisms directed to pigs or humans taking into consideration the anatomical, physiological, and immunological similarities of the gastrointestinal tract of both hosts. Among the strains evaluated in PIE cells, *L. delbrueckii* subsp. *delbrueckii* TUA4408L, an strain isolated from a Japanese traditional pickle fermented food, was able to reduce the expression of proinflammatory cytokines after TLR4 activation. This immunomodulatory activity was related to its ability to diminish the activation of nuclear factor κB (NF-κB) and MAPK pathways in a TLR2-dependent manner (11). Moreover, the EPS of the TUA4408L strain was fractionated into acidic (APS) and neutral (NPS) fractions and, it was demonstrated that both APS and NPS differentially regulated the expression of IL-6, IL-8, and monocyte chemoattractant protein-1 (MCP-1) in PIE cells after the stimulation with the TLR4 agonist. Both APS and NPS reduced the activation of NF-κB, ERK-MAPK, and p38-MAPK pathways while NPS also decreased the activation of JNK-MAPK pathway (11).

Recently, substantial progress has been achieved in genomic sequencing of LAB and now several collections of lactobacilli genomes are available. Until the writing of this manuscript was completed, fifty complete and draft genomes belonging to the *L. delbrueckii* species were published in the NCBI database, including *L. delbrueckii* subsp. *bulgaricus*, subsp. *lactis*, subsp. *delbruekii*, subsp. *jakobsenii*, subsp. *indicus*, and subsp. *sunkii*. Among them, two complete and two draft genomes are available for different strains of *L. delbrueckii* subsp. *delbruekii*. Therefore, few genomic analyzes have been performed with this species of LAB.

In this work, we further advanced in the characterization of the immunomodulatory properties of *L. delbrueckii* TUA4408L and its EPSs. Whole genome sequencing allowed the analysis of the general features of *L. delbrueckii* TUA4408L genome as well as the characterization of its EPS gene cluster. In addition, studies in PIE cells demonstrated that the TUA4408L strain and its EPSs are able to modulate the antiviral innate immune response triggered by TLR3 activation. Moreover, we demonstrated here for the first time that *L. delbrueckii* TUA4408L and its EPSs are capable of improving the resistance of intestinal epithelial cells against rotavirus infection by reducing viral replication and differentially modulating antiviral response.

## Materials and methods

### Microorganisms

*Lactobacillus delbrueckii* subsp. *delbrueckii* TUA4408L was isolated from sunki-zuke, a japanease traditional pickle fermented in salt-free conditions. The TUA4408L strain was maintained in deMan-Rogosa-Sharp (MRS) medium at 4°C for further usage. For experiments, TUA4408L strain was propagated in soy milk at 43°C for 16 h, washed with PBS and diluted in Dulbecco's modified Eagle's medium (DMEM, Invitrogen corporation, Carlsbad, CA). The EPSs from *L. delbrueckii* TUA4408L were extracted and fractionated by the method of Kitazawa et al. (12). The acid (APS) and neutral (NPS) EPSs were fractionated by ion-exchange chromatography as described previously (11).

Rotavirus strain UK was used in challenge experiments considering its ability to efficiently replicate in PIE cells, as described previously (13). Rotavirus UK was treated with 10 μg/ml of trypsin (Sigma, Type I) at 37°C for 30 min, and then inoculated onto confluent MA104 cells. After 1 h of absorption, the inoculum was removed and the cells were incubated with serum-free MEM (1 μg/ml of trypsin) at 37°C. After the cytopathic effect reached more than 80% of cells, three rounds of freezing and thawing were performed to harvest the culture supernatant. The virus stock was stored at −80°C for further experiments.

### Bioinformatic analysis

*L. delbrueckii* subsp. *delbrueckii* TUA4408L genome was sequenced with the PacBio sequencing platform and genome assembly was performed using HGAP 3.0 (14), with default options and the Minimum Seed Read Length was adjusted to 2,000. *L. delbrueckii* TUA4408L genome annotation was carried out using NCBI Prokaryotic Genome Annotation Pipeline (15). Further annotation was obtained by using the SEED-based automated annotation system provided by the RAST server (16). Genome sequencing project was deposited in GenBank under accession number CP021136.

Circular genome maps were generated using the CGView Server (17) based on the information generated by the genome annotation. Bioinformatics analyses of the TUA4408L genome included the use of Antibiotic Resistance Genes Database (ARDB) (18), and Comprehensive Antibiotic Resistance Database (CARD) (19) to evaluate antibiotic resistance genes, PHAge Search Tool (PHAST) and IslandViewer software were used to identify prophages and genomics islands, respectively (20, 21). Clustered Regularly Interspaced Short Palindromic Repeat (CRISPR)-Cas arrays were identified in bacterial genome using CRISPRfinder (22). The presence and type of bacteriocin genes was evaluated using BAGEL4 software and BLASTx algorithm.

Two phylogenetic trees were constructed based on multilocus sequence typing (MLST), and the 16S rRNA gene, respectively. Seven different housekeeping genes of *L. delbrueckii* were used to construct the MLST phylogenetic tree: *fusA, gyrB, hsp60, ileS, pyrG, recA*, and *recG* (23). The 16S rRNA gene sequences and the corresponding nucleic acid sequences from the seven genes for MLST were respectively aligned with those of the most closely related species using the multiple alignment program MUltiple Sequence Comparison by Log- Expectation (MUSCLE) (24), a bioinformatic tool included in MEGA7 (25).

For comparative genomic analysis, the complete genomes of *Lactobacillus delbrueckii* subsp. *delbrueckii* TUA4408L, KCTC 13731, DSN 20074, NBRC 3202, and KACC 13439 strains were used. Nucleotide sequences were obtained from GenBank and genomes were reannotated using Prokka (15). Roary 3.11.2, is a high-speed stand-alone pan genome pipeline, which takes annotated assemblies in GFF3 format produced by Prokka (26) and calculates the pan genome was used.

### Cell culture

The PIE cell line was originally established at Tohoku University from intestinal epithelia of unsuckled neonatal swine (10). PIE cells were maintained in DMEM medium supplemented with 10 % fetal calf serum (FCS), penicillin (100 mg/ml), and streptomycin (100 U/ml) at 37°C in a humidified atmosphere of 5% CO_2_. PIE cells grow rapidly without any transformation or immortalization. Their proliferative capability diminishes after 50 passages in culture. Therefore, PIE cells in this study had between 20th and 40th passages. The cultures were passaged routinely after reaching confluence of 80–90%.

### Analysis of antiviral immunity in PIE cells

PIE cells were seeded at 3.0 × 10^4^ cells in 12 well type I collagen coated plates and incubated at 37°C, 5% CO_2_. After 3 days of culturing period, 1 ml of DMEM containing either *L. delbrueckii* (5 × 10^7^ cells/ml), APS or NPS (100 ug/ml) was added. Cells were incubated at 37°C, 5% CO_2_ for 48 h. PIE cells were washed three times with fresh medium to eliminate lactobacilli or EPSs and subsequently stimulated with 10 ug/ml of poly(I:C) (Sigma Aldrich, USA) for 12 h. The expressions of interferon (IFN)-β, IL-6, IL-8, MCP-1, TNF-α, RNaseL, Myxovirus resistance gene A (MxA), retinoic acid-inducible gene I receptor (RIG-I), and TLR3 were quantified by qPCR as described below.

### Analysis of TLR negative regulators in PIE cells

In order to analyze the expression of negative regulators of TLR signaling, PIE cells were seeded at 3.0 × 10^4^ cells/well in 12 well type I collagen coated plates and cultured at 37°C, 5% CO_2_ for 3 days. Then, 1 ml of DMEM containing either *L. delbrueckii* (5 × 10^7^ cells/ml), APS or NPS (100 ug/ml) was added. After 48 h of stimulation, PIE cell were stimulated with poly(I:C) as described above, for 3, 6, and 12 h. The expression of TLR negative regulators single immunoglobulin IL-1-related receptor (SIGIRR), Toll interacting protein (Tollip), zinc finger protein A20 (A20), B-cell lymphoma 3-encoded protein (Bcl-3), mitogen-activated protein kinase-1 (MKP-1), and interleukin-1 receptor-associated kinase M (IRAK-M) were quantified by qPCR as described below.

### RNA extraction and qPCR

The total RNA was isolated by using TRIzol reagent (Invitrogen). The purity and quantity of RNA was analyzed by Nano drop spectrophotometer ND-1000 UV-Vis (NanoDrop Technologies, USA). The quantified RNA (500 ng) was used to synthesize cDNA by Thermal cycler (BIO-RAD, USA). The reaction mixtures (10 μl) were prepared using Quantitect reverse transcription (RT) kit (Qiagen, Tokyo, Japan) according to the manufacturer instructions. The qPCR was performed in a 7300 real-time PCR system (Applied Biosystems, Warrington, UK) with platinum SYBR green (qPCR supermix uracil-DNA glycosylase with 6-carboxyl-X-rhodamine, Invitrogen). The total volume of reaction mixture was 10 μl, which contained 2.5 μl of cDNA, and 7.5 μl of master mix that included RT enzyme, SYBR green, forward and reverse primers (1 pmol/μl). The reaction cycles were performed first at 50°C for 5 min; followed by 95°C for 5 min; then 40 cycles at 95°C for 15 s, at 60°C for 30 s and at 72°C for 30 s. According to the minimum information for publication of qPCR experiments guidelines, β-actin was used as a housekeeping gene because of its high stability across porcine various tissues (27, 28). Expression of β-actin was used to normalize cDNA levels for differences in total cDNA levels in the samples. Primers were described previously (11, 13).

### Western blotting analysis

PIE cells were seeded (1.8 × 10^5^ cells/dish) in 60 mm dishes and incubated at 37°C, 5% CO_2_ for 3 days. The confluent PIE cells were stimulated with lactobacilli or EPSs as described above. After stimulation, PIE cells were washed three times with fresh DMEM medium, treated with 10 ug/ml of poly(I:C) and studied in different time intervals (0, 30, 60, 120, 180, and 240 min). Then, PIE cells were washed three times with PBS and the harvested cells were lysed by adding 200 ul of CellLytic M cell lysis reagent (Sigma-Aldrich, St. Louis, MO, USA), containing protease inhibitors (Complete mini, PhosSTOP, Roche, Mannheim, Germany). Afterwards, the lysed cells were transferred to fresh Eppendorf tubes (1.5 ml), kept on ice for 5–10 min and sonicated three times at 50% for 3 sec and, centrifuged at 150 × 100 rpm for 5 min. Then, the concentration of protein in the resulting supernatants was estimated by using bicinchoninic acid (BCA) assay kit (Thermo Scientific, Pierce, Rockford, IL).

For western blot analysis, the protein samples (8 ug/sample) were loaded on 10% SDS-polyacrylamide gels and the electrophoretically separated proteins were transferred to a nitrocellulose membrane (Trans-Blot TurboTM, BIO-RAD). The membranes were cut and incubated with blocking buffer for 1–2 h before incubating with primary and secondary antibodies. TNF receptor-associated factor (TRAF3), Interferon regulatory factor 3 (IRF-3), p38 and the nuclear factor kappa B inhibitor alpha (IkBα) were evaluated using TRAF3 antibody (Cat. #4729), Phospho-IRF3 (Ser396) (4D4G, Cat. #4947) rabbit antibody, Phospho-p38 mitogen-activated protein kinase (Thr180/Tyr182) antibody (p-p38, Cat. #9211); and I kappaB-alpha antibody (IkBa, Cat. #9242) from Cell Signaling Technology (Beverly, MA, USA) at 1,000 times dilution of their original concentration, overnight at room temperature. Then, membranes were washed with TBS-T buffer and incubated with anti-rabbit IgG, AP-linked antibody (Cat. #7054) for 1–2 h at room temperature. After washing with TBS-T buffer, the membranes were spread with 200 ul of ECF substrate (GE Healthcare Japan Co., Tokyo, Japan) to detect optical protein bands and photographed by blot analyzer (Quantity One W409). The exhibited proteins bands were estimated from the peak area of densitogram by using Image J software (National Institute of Health, Bethesda, MD, USA). To detect each total protein, the same membranes were incubated in stripping solution for 10 min (Western Blot Re-Probe Kit, #JZ-008, Jacksun Easy Biotech, Inc., New York, USA), blocked (1–2 h), and subsequently incubated with IRF3 (D83B9, Cat. #4302) antibody, p38 MAPK antibody (p38, Cat. #9212), and β-actin (13E5, Cat. #4970) rabbit antibody, from Cell Signaling Technology.

### Role of TLRs in the immunomodulatory activities of *L. delbrueckii* TUA4408L

The role of TLR2 and TLR4 in the immunomodulatory activities of *L. delbrueckii* TUA4408L and its EPSs were analyzed by using blocking experiments. Unlabeled anti-porcine TLR2 and TLR4-rabbit IgG antibodies (Biolegend, San Diego, CA) were used to block TLRs expression in PIE cells as described previously (11). Briefly, PIE cells were cultured (3.0 × 10^4^ cells/ml) in 12 well type I collagen coated plates at 37°C, 5% CO_2_. After 3 days, the confluent cells were incubated with unlabeled anti-porcine TLR2 or TLR4 IgG antibodies (200 ng/ml) for 12 h. After treatment with blocking antibodies, PIE cells were treated with *L. delbrueckii* TUA4408L or its EPSs as described above. Finally, PIE cells were washed three times with fresh medium and subsequently stimulated with poly(I:C) (10 ug/ml) for 12 h at 37°C, 5% CO_2_. The expression of IFN-β, IL-6, IL-8, MCP-1, and TNF-α were examined by qPCR as described before.

### Rotavirus infection

PIE cells were plated (5.0 × 10^3^) in 96 well type I collagen coated microplate (SUMILON, Tokyo, Japan) and incubated at 37°C, 5 % CO_2_. One ml of DMEM containing either *L. delbrueckii* (5 × 10^7^ cells/ml), APS or NPS (100 ug/ml) was added. After 48 h incubation, the cells were treated with trypsin-activated rotavirus UK, and incubated at 37°C, 5% CO_2_. At hour 16 post-inoculation, PIE cells were fixed after removal of the inoculums and the infected virus titer were analyzed by immunofluorescence staining as described previously (13). Infection rate of UK rotavirus in PIE cells is 4.6 log10 FFU/0.1 ml (13). In addition, the expression of IFN-β, MxA, RIG-I, TLR3, IL-6, IL-8, and MCP-1 were quantified by qPCR.

### Statistical analysis

Statistical analyses were performed using the GLM and REG procedures available in the SAS computer program (SAS, 1994). Comparisons between mean values were carried out using one-way analysis of variance and Fisher's least-significant-difference (LSD) test. For these analyses, *P* values of < 0.05 were considered significant.

## Results

### General features of *L. delbrueckii* TUA4408L genome

**Figure 1:**
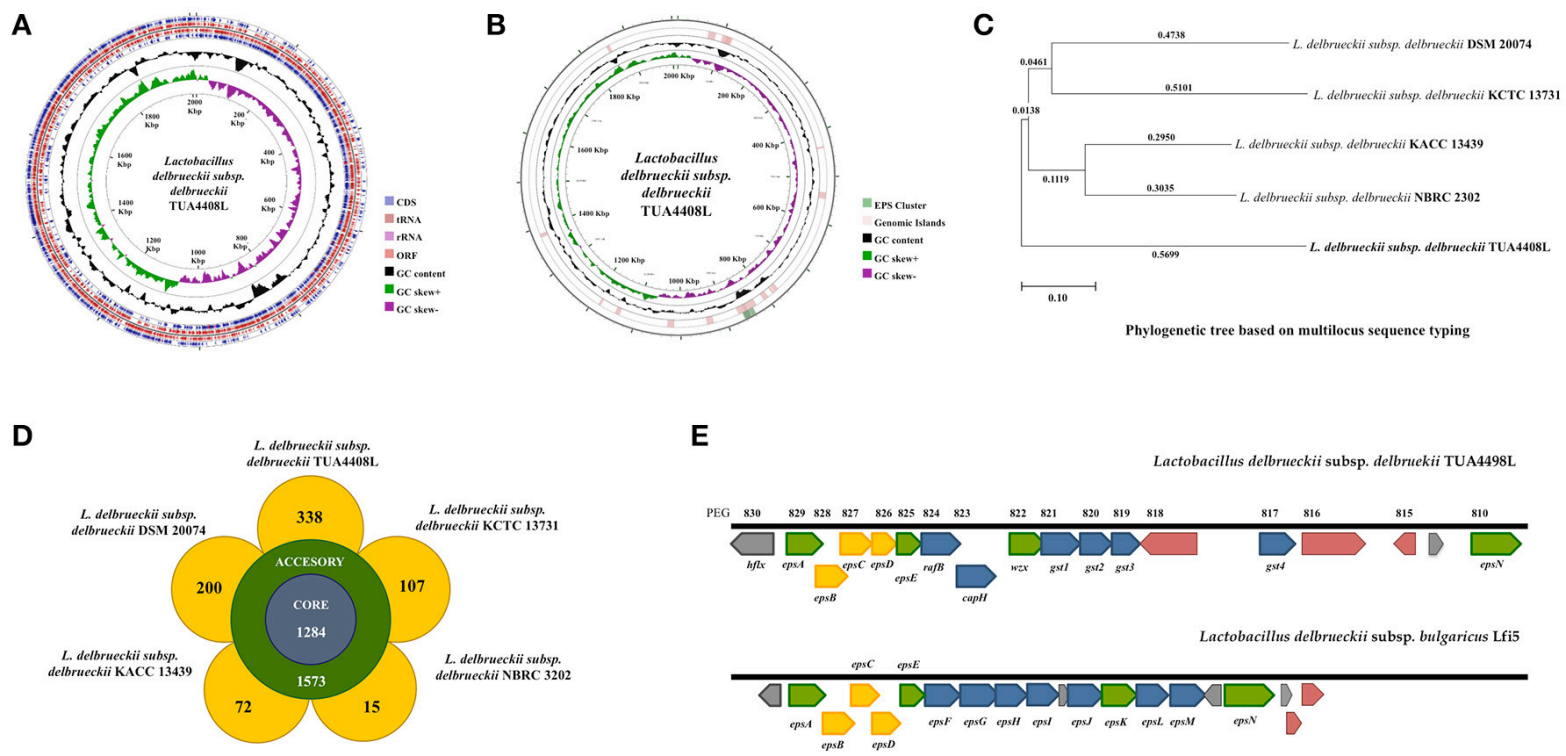
Genomic analysis of the exopolysaccharide (EPS) producing strain *Lactobacillus delbrueckii* subsp. *delbrueckii* TUA4408L. **(A)** Genome was sequenced with the PacBio sequencing platform, assembled with HGAP 3.0, and annotated with NCBI Prokaryotic Genome Annotation Pipeline. Circular genome map of the TUA4408 strain was generated with the CGView Server. Genome sequencing project was deposited in GenBank under accession number CP021136. **(B)** EPS gene cluster and genomic islands are highlighted in the circular genome map of the TUA4408 strain was generated with the CGView Server. **(C)** Phylogenetic tree of *Lactobacillus delbrueckii* subsp. *delbrueckii* constructed based on multilocus sequence typing (MLST) genes. Seven different housekeeping genes of *L. delbrueckii* were used to construct the MLST phylogenetic tree including *fusA, gyrB, hsp60, ileS, pyrG, recA*, and *recG*. **(D)** Pangenome analysis of *L. delbrueckii* subsp. *delbrueckii*. The complete genomes of TUA4408L, KCTC 13731, DSN 20074, NBRC 3202, and KACC 13439 strains were used. Nucleotide sequences were obtained from GenBank and genomes were reannotated using Prokka. Roary 3.11.2 pangenome pipeline was used for analysis. **(E)** Analysis of the EPS gene cluster of TUA4408L strain.Comparison of EPS gene cluster among *L. delbrueckii* subsp. *delbrueckii* TUA4408L and *L. delbrueckii* subsp. *bulgaricus* Lfi5. The predicted functions of genes are indicated by colors as follow: assembly (green), modulation (yellow), and glycosyltransferase (blue).

The genome of *L. delbrueckii* TUA4408L was assembled into a single circular chromosome composed of 2,012,440 bp with 49.9% G+C content (Figure 1A; Table 1). Genome sequencing also revealed that TUA4408L strain is a plasmid-free bacterium. A total of 2,029 genes (1,755 protein-coding genes), 27 rRNA (including 5S, 16S, and 23S genes), 95 tRNA, 3 ncRNA, and 222 pseudo genes were found in the circular chromosome of TUA4408L strain (Table 1).

**Table 1:** Comparison of the general genome features of sequenced *Lactobacillus delbrueckii* subsp. *delbrueckii*.

**Attribute**	***Lactobacillus delbrueckii* subsp. *delbrueckii* TUA4408L**	***Lactobacillus delbrueckii* subsp. *delbrueckii* KCTC 13731**	***Lactobacillus delbrueckii* subsp. *delbrueckii* DSM 20074**	***Lactobacillus delbrueckii* subsp. *delbrueckii* ACC 13439**	***Lactobacillus delbrueckii* subsp. *delbrueckii* NBRC 3202**
GenBank accession	CP021136	CP018216	CP018615	LHPL00000000	BEWJ00000000
Genome size (bp)	2,012,440	1,910,506	1,953,716	1,766,190	1,787,093
DNA GC content (%)	49.9	50.0	49.6	50.1	50.3
Level	Complete genome	Complete genome	Complete genome	Conting	Conting
Plasmid	Not reported	Not reported	Not reported	Not reported	Not reported
rRNA genes (5S, 16S, 23S)	27 (9, 9, 9)	24 (8, 8, 8)	27 (9, 9, 9)	4 (0, 3, 1)	3 (1, 1, 1)
tRNA genes	95	84	95	65	50
Other ncRNA genes	3	3	3	Not reported	3
Proteins	1,755	1,617	1,587	1,521	1,599
Predicted genes	2,029	1,921	1,984	1,804	1,816
Pseudo genes	149	193	272	214	161
CRISPR Arrays	1	1	Not reported	Not reported	1

By using the PHage Search Tool (PHAST), *L. delbrueckii* TUA4408L genome was predicted to have two incomplete prophage regions, located at positions 136643–147495 (10.8 Kbp) and 474436–498733 (24.2 Kbp). In addition, genomics islands were found in different blocks of TUA4408L genome (Figure 1B). The *in silico* analysis of the distribution, the presence and type of bacteriocin genes was evaluated in *L. delbrueckii* TUA4408L using BAGEL4 software and BLASTx algorithm. Analysis of genome sequence of the TUA4408L strain revealed the presence of two ORF encoding enterolysin A, a cell wall-degrading bacteriocin widely distributed among *Enterococcus strains* (29, 30). This gene is also detected among *L. delbrueckii* genomes with high percentage of identity (data not shown). Antibiotic resistance genes were not found in the TUA4408L strain by using ARDB and CARD. The genome of *L. delbrueckii* TUA4408L contains CRISPR loci and Cas proteins that provide sequence-specific protection against foreign DNA (31). In the TUA4408L genome we found a type IC CRISPR–Cas system constituted by *cas3, cas5, cas8c, cas7, cas4, cas1, cas2*, and a CRISPR array with 33 short direct repeats and 18 spacers.

RAST analysis indicated that *L. delbrueckii* TUA4408L has a remarkable potential to metabolize sugars since its genome contains several genes related to chitin, N-acetylglucosamine, sucrose, maltose, maltodextrin, mannitol, and D-ribose utilization. In addition, genes for trehalose, lactose and galactose uptake and utilization, as well as trehalose biosynthesis were found in the genome.

We also compared the genome of the TUA4408L strain with the available genomes of other *L. delbrueckii* subsp. *delbrueckii* strains including DSM 20074, NBRC 2302, KCTC 13731, and KACC 13439 (Figures 1C,D). Phylogenetic tree constructed on multilocus sequence typing (MLST) showed that *L. delbrueckii* TUA4408L clustered separated from the other sequenced strains (Figure 1C). In order to obtain better insight into the specific features of *L. delbrueckii* TUA4408L, we performed a pangenome analysis with the TUA4408L genome and the four genomes mentioned before. We found that 1,284 genes belong to the coregenome sheared by the five strains evaluated while 1,573 genes belong to the accessory pangenome (Figure 1D). A relative low number of unique genes were found for NBR 2303 (15 genes), and KACC 13439 (72 genes) strains, probably because they are not completely sequenced. *L. delbrueckii* KCCT 13731 and DSM 20074 strains had 107 and 200 unique genes, respectively (Figure 1D). Interestingly, *L. delbrueckii* TUA4408L had 338 unique genes including 136 proteins with known functions and 202 hypothetical proteins.

Among the unique genes present in the *L. delbrueckii* TUA4408L genome, we found glycosyltransferases involved in EPS biosynthesis (discussed below). In addition, several genes involved sugar metabolism were found in the TUA4408L strain but not in the other *L. delbrueckii* subsp. *delbrueckii* strains analyzed (Supplemental Table 1). The phosphoenolpyruvate-dependent sugar phosphotransferase system (PTS), a major carbohydrate active transport system, catalyzes the phosphorylation of incoming sugar substrates concomitant with their translocation across the cell membrane. Several PTS genes were found in the group of unique genes from the TUA4408L strain including those involved in the transport of glucose (*crr3*), lactose (*lacF*), mannose (*manX, manZ*), oligoglucomannans such as cellobiose (*celA, celD, bglK*) and mannobiose (*gmuC*), lichenan (*licA, licC*), maltose (*malP*), the chitin disaccharide N,N'-diacetylchitobiose (*chbB*), and L-sorbose (*sorA, sorB*) (Supplemental Table 1). Transporters for trehalose (*sugC*), ribose (*rbsB*), and L-arabinose (*araQ*), genes involved in the utilization of different sugars including *bglA* that plays a major role in the utilization of arbutin or salicin, and *levS* that participates in starch and sucrose metabolism, as well as genes involved in the biosynthesis of glycogen (*glgA, glgB, glgC*, and *glgD*), galactofuranose glycoconjugates (*glf*), and sucrose (*inuJ*) were also found in the group of unique genes of TUA4408L.

### Characterization of EPS gene cluster of *L. delbrueckii* TUA4408L

As expected, all the genes necessary for EPS production were found in the genome of *L. delbrueckii* TUA4408L (Figure 1E). In LAB, it has been described that a typical EPS gene cluster consists of five highly conserved genes *epsA, epsB, epsC, epsD*, and *epsE*, and a variable region, which includes the genes for the polymerase *wzy*, the flippase *wzx*, and a variable number of glycosyltransferases and other polymer-modifying enzymes [reviewed in (2)]. This type of EPS gene cluster structure has been described for *L. delbrueckii* subsp. *bulgaricus* Lfi5 (32). An 18Kpb DNA region containing 14 genes, designated *epsA* to *epsN*, was isolated by genomic DNA library screening and inverted PCR for the Lfi5 strain (Figure 1E). Similarly, *epsA* to *epsE* genes are present in the genome of *L. delbrueckii* TUA4408L (Figure 1E), which show high percentages of identity with those described in the Lfi5 strain (Supplemental Table 2). Therefore, putative functions for these EPS gene products in *L. delbrueckii* TUA4408L could be assigned by sequence similarities: *epsA* would be a positive regulator of the *eps* operon, the *epsBCD* would be a phosphoregulatory system, while *epsE* would be the phosphoglucosyltransferase initiating the biosynthesis of EPS. The polymerase *wzy* (*epsK*) and the flippase *wzx* (*epsN*) are also found in the EPS cluster of *L. delbrueckii* TUA4408L (Figure 1E). In addition, seven genes coding for glycosyltransferases are present in the TUA4408L genome including members of the glycosyltransferases superfamilies GTB, glyco-transf-2-3, Caps-Shynth and Core-2/I-branching enzyme (Supplemental Table 3). Most of the genes in the EPS cluster of *L. delbrueckii* TUA4408L are tightly coupled (Figure 1E); significant intergenic gaps only exist between *gst3* and *gst4* (3,989 bp) and between *gst4* and *epsN* (5,754 bp). However, predictable transcription terminators or consensus promoter sequences were not found in these gaps.

### *L. delbrueckii* TUA4408L and its EPSs improve resistance of PIE cells to rotavirus infection and differentially modulate antiviral immune response

**Figure 2:**
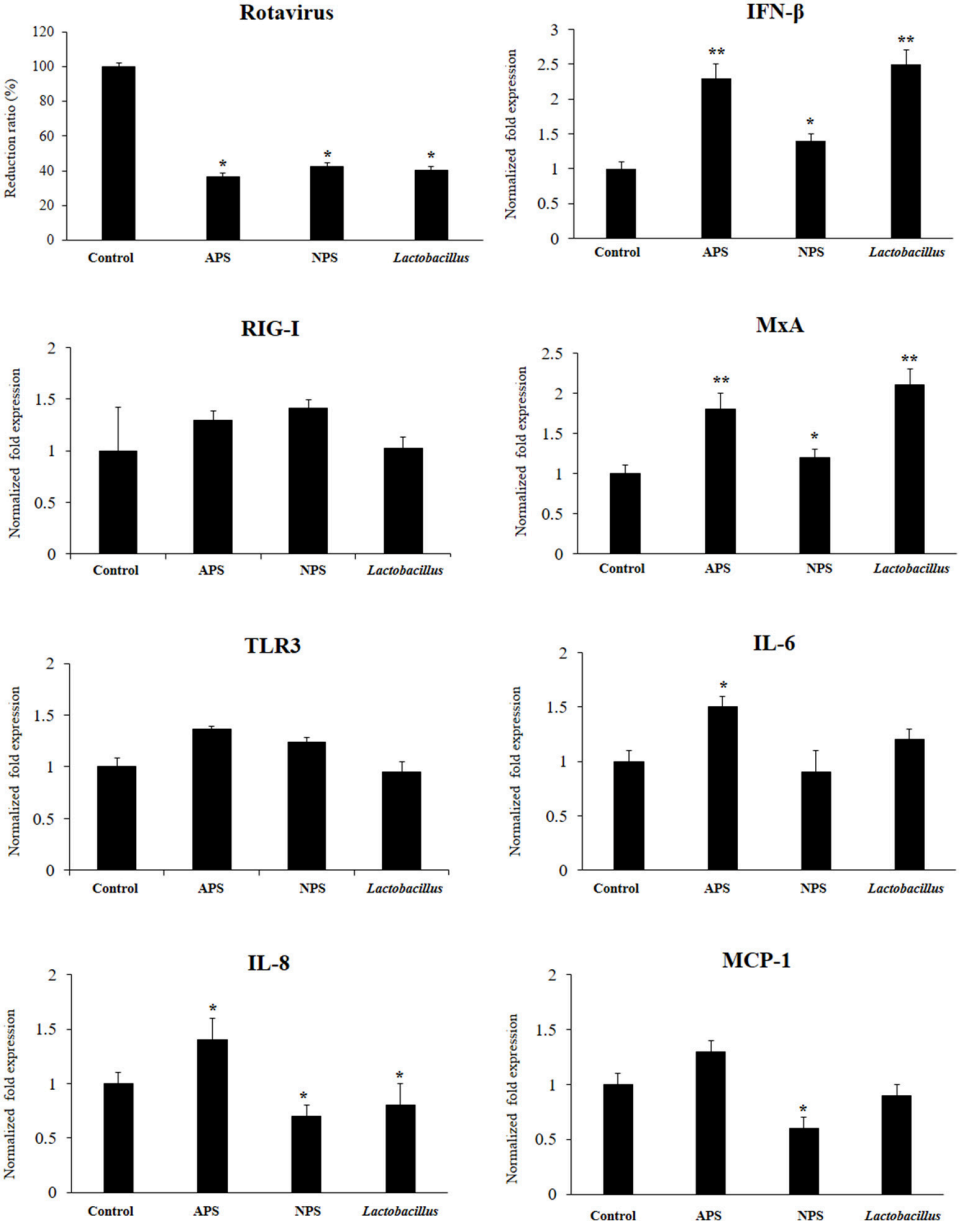
Antiviral activity of exopolysaccharides (EPS) from *Lactobacillus delbrueckii* subsp. *delbrueckii* TUA4408L in porcine intestinal epithelial (PIE) cells. EPS was fractionated into neutral (NSP) and acidic (APS) EPS by standard chromatographic methods. PIE cells were treated with lactobacilli, APS, NPS, or medium for 48 h and then infected with rotavirus. The virus titers were analyzed by immunofluorescence staining 16 h after challenge. The expression of interferon β (IFN-β), MxA, Toll-like receptor (TLR)-3, retinoic acid-inducible gene I receptor (RIG-I), interleukin 6 (IL-6), IL-8, and monocyte chemoattractant protein-1 (MCP-1) was evaluated 16 h after rotavirus challenge. The results are expressed as mean ± SD and represent data from three independent experiments (*n* = 3 in each experiment). Asterisks indicate significant differences when compared to rotavirus-challenged control PIE cells (*P < 0.05 and **P < 0.01).

We aimed to evaluate the capacity of EPSs from *L. delbrueckii* TUA4408L to improve the resistance against rotavirus infection in PIE cells that are susceptible to the infection with this pathogen (27). PIE cells were stimulated with *L. delbrueckii*, APS or NPS for 48 h and then challenged with rotavirus strain UK. As we described previously, rotavirus UK efficiently replicated in PIE cells (Figure 2). Of interest, the three treatments *L. delbrueckii*, APS and NPS were able to significantly reduce rotavirus replication in PIE cells (Figure 2).

The innate antiviral immune response triggered by rotavirus infection was also evaluated. The challenge of PIE cells with rotavirus significantly increased the expression of IFN-β, MxA, IL-6, IL-8, and MCP-1 when compared to untreated control cells, in line with our previous findings (27). Treatment of PIE cells with *L. delbrueckii* TUA4408L, NPS or APS significantly increased the expression of IFN-β and MxA when compared to controls (Figure 2). However, APS and *L. delbrueckii* were more efficient to improve the expression of both antiviral factors than NPS. No significant differences were observed between control and treated PIE cells when the expression levels of RIG-I or TLR3 were evaluated after rotavirus infection (Figure 2). When the inflammatory cytokines and chemokines were analyzed, it was observed that each treatment induced a characteristic change in IL-6, IL-8, and MCP-1 expression in PIE cells after rotavirus infection (Figure 2). APS significantly enhanced the expression of IL-6 and IL-8, while NPS reduced the expression of IL-8 and MCP-1 in infected PIE cells when compared to untreated controls. In addition, *L. delbrueckii* TUA4408L diminished IL-8 expression in in PIE cells afterrotavirus infection (Figure 2).

### *L. delbrueckii* TUA4408L and its EPSs differentially modulate the TLR3-triggered cytokine response in PIE cells

**Figure 3:**
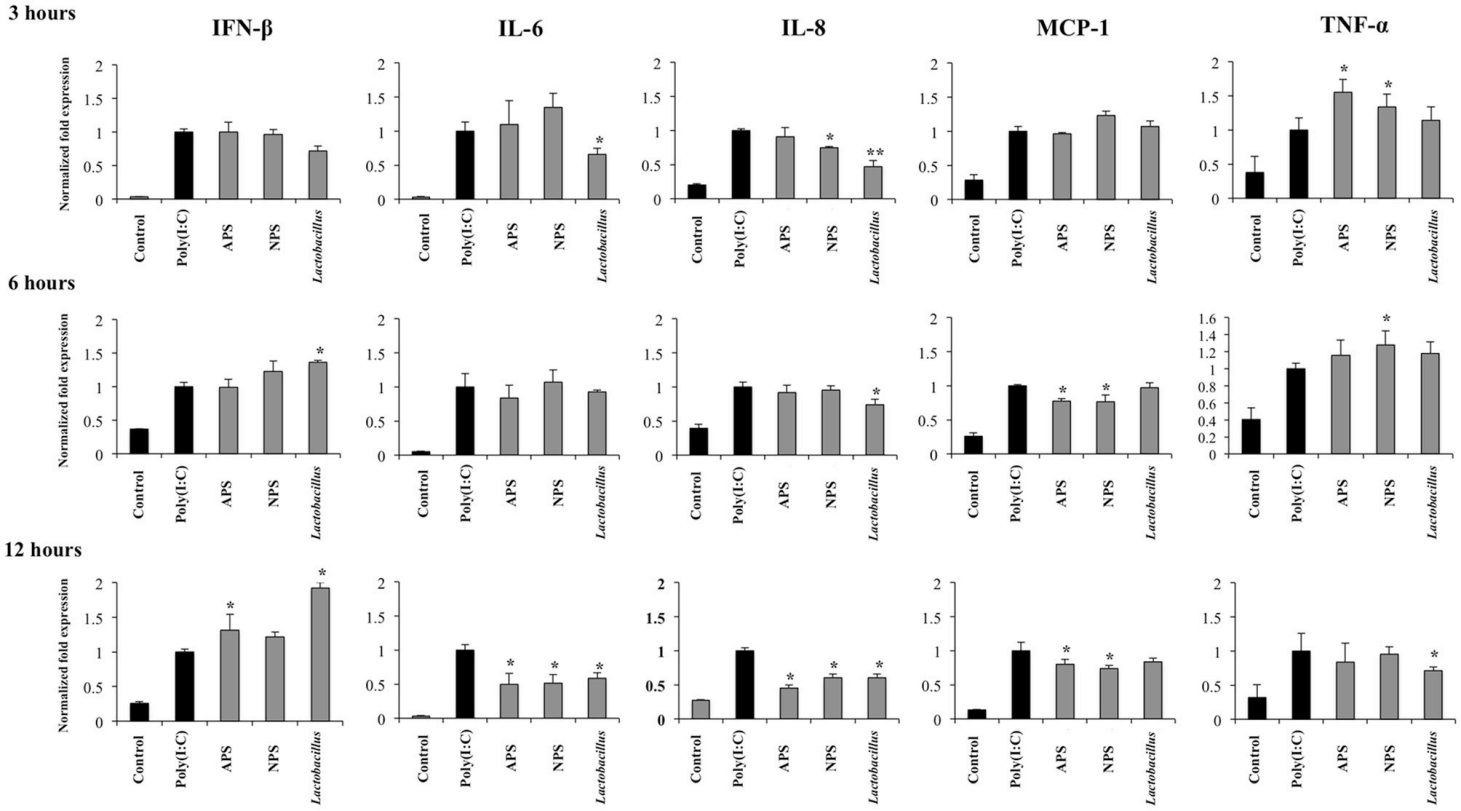
Immunomodulatory activity of exopolysaccharides (EPS) from *Lactobacillus delbrueckii* subsp. *delbrueckii* TUA4408L in porcine intestinal epithelial (PIE) cells. EPS was fractionated into neutral (NSP) and acidic (APS) EPS by standard chromatographic methods. PIE cells were treated with lactobacilli, APS, NPS or medium for 48 h and then stimulated with the Toll-like receptor (TLR)-3 agonist poly(I:C). PIE cells without poly(I:C) stimulation were used as controls. The expression of interferon β (IFN-β), interleukin 6 (IL-6), IL-8, monocyte chemoattractant protein-1 (MCP-1), and tumor necrosis factor (TNF-α) was evaluated at 3, 6, and 12 h after poly(I:C) challenge. The results are expressed as mean ± SD and represent data from three independent experiments (n = 3 in each experiment). Asterisks indicate significant differences when compared to poly(I:C)-challenged control PIE cells (*P < 0.05, **P < 0.01).

Considering the ability of EPSs from *L. delbrueckii* TUA4408L to improve innate immune response against rotavirus, we next evaluated their influence in the innate antiviral immune response triggered by TLR3, which is known to be the main pattern recognition receptor (PRR) activated by this pathogen (33). For this purpose, PIE cells were stimulated with *L. delbrueckii*, APS, or NPS for 48 h and then challenged with the TLR3 ligand poly(I:C). The expression of IFN-β, IL-6, IL-8, MCP-1, and TNF-α were determined at different time points after poly(I:C) stimulation as shown in Figure 3. Poly(I:C) significantly increased the expression of all the inflammatory factors evaluated when compared to untreated control cells, in line with our previous findings (34, 35). Treatment of PIE cells with *L. delbrueckii* TUA4408L significantly increased the expression of IFN-β at hours 6 and 12 post-poly(I:C) stimulation. APS also increased the expression of IFN-β on hour 12 while NPS did not induce changes in the expression of this type I IFN (Figure 3).

*L. delbrueckii* TUA4408L significantly reduced the expression of IL-6 and IL-8 early at hour 3 after poly(I:C) stimulation, and TNF-α at hour 12 (Figure 3). No effect on MCP-1 expression was observed in lactobacilli-treated cells. APS and NPS treatments significantly reduced the expression of IL-6, IL-8, and MCP-1 at hour 12 after poly(I:C) stimulation, while the both treatments enhanced the levels of TNF-α mRNA at hour 3 (Figure 3).

**Figure 4:**
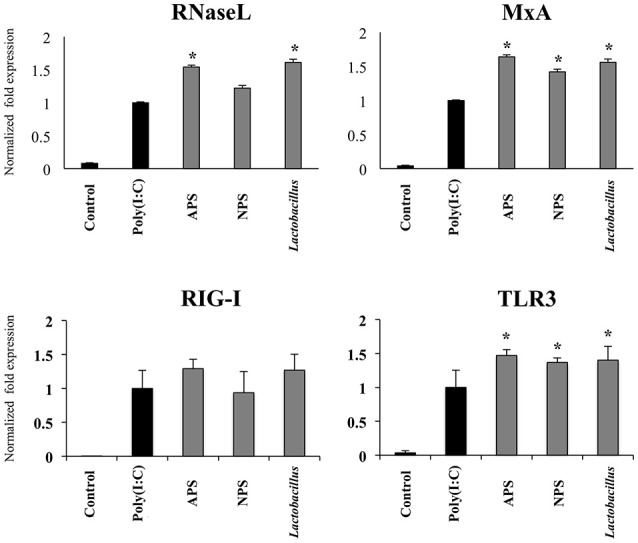
Immunomodulatory activity of exopolysaccharides (EPS) from *Lactobacillus delbrueckii* subsp. *delbrueckii* TUA4408L in porcine intestinal epithelial (PIE) cells. EPS was fractionated into neutral (NSP) and acidic (APS) EPS by standard chromatographic methods. PIE cells were treated with lactobacilli, APS, NPS or medium for 48 h and then stimulated with the Toll-like receptor (TLR)-3 agonist poly(I:C). PIE cells without poly(I:C) stimulation were used as controls. The expression of the pattern recognition receptors TLR3 and RIG-I was evaluated 12 h after poly(I:C) challenge and the antiviral factors RNAseL and MxA 24 h after stimulation. The results are expressed as mean ± SD and represent data from three independent experiments (*n* = 3 in each experiment). Asterisks indicate significant differences when compared to poly(I:C)-challenged control PIE cells (*P < 0.05).

Taking into consideration the ability of *L. delbrueckii* and its EPSs to differentially modulate the expression of IFN-β in PIE cells after TLR3 activation, we next evaluated the expression of factors involved in antiviral defenses that are induced by this type I IFN including RNaseL, MxA, RIG-I, and TLR3 (Figure 4). The three treatments improved the expression of TLR3 (Figure 4) and MxA (data not shown) in poly(I:C)-treated PIE cells while no changes were observed in RIG-I (Figure 4) and RNaseL (data not shown) mRNA levels at hour 12 after poly(I:C) stimulation. When MxA and RNaseL were evaluated at hour 24 after poly(I:C) challenge, we found that treatments improved the expression of MxA while *L. delbrueckii* TUA4408L and APS increased RNaseL (Figure 4).

### *L. delbrueckii* TUA4408L and its EPSs differentially modulate TLR3 signaling pathway in PIE cells

**Figure 5:**
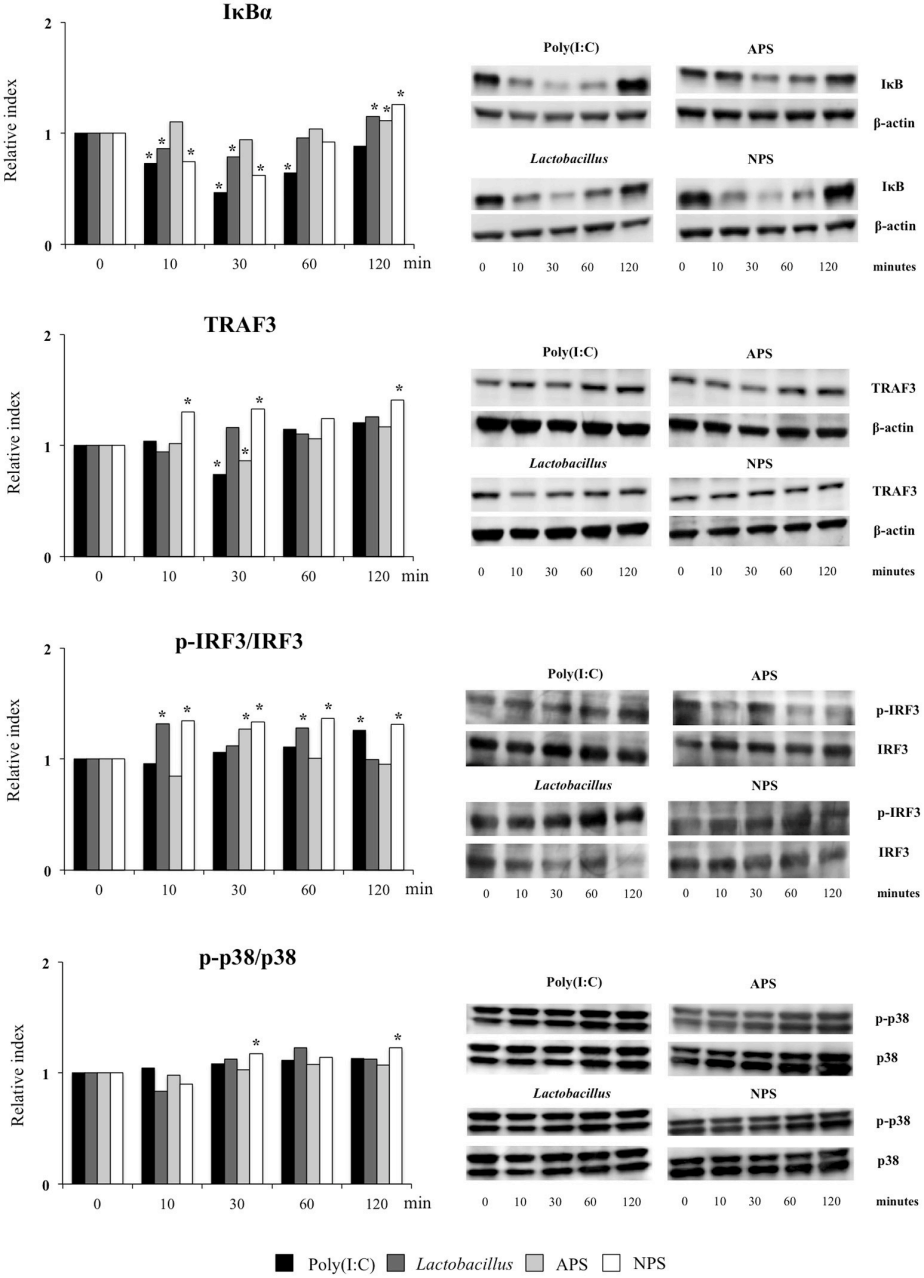
Effect of exopolysaccharides (EPS) from *Lactobacillus delbrueckii* subsp. *delbrueckii* TUA4408L on signaling pathways in porcine intestinal epithelial (PIE) cells. EPS was fractionated into neutral (NSP) and acidic (APS) EPS by standard chromatographic methods. PIE cells were treated with lactobacilli, APS, NPS or medium for 48 h and then stimulated with the Toll-like receptor (TLR)-3 agonist poly(I:C). PIE cells without poly(I:C) stimulation were used as controls. The proteins from lysed cells were extracted at the indicated time points and separated by SDS-PAGE. Western-blot was performed to quantify TRAF3, p-IRF3/IRF3/, p-p38/p38, and IκBα levels. Intensities of proteins bands were calculated from peak area of densitogram by using image J software. Three independent experiments, one representative experiment is shown. Asterisks indicate significant differences when compared to time 0 for each group (*P < 0.05).

The TLR3 signaling pathway in PIE cells was studied by evaluating TRAF3, p-IRF3/IRF3, p-p38/p38, and IκBα by western blot analysis for 120 min after poly(I:C) stimulation (Figure 5).

Challenge of PIE cells with poly(I:C) significantly reduced the levels of the IκBα between 10 and 60 min, indicating the activation of the NF-κB pathway (Figure 5). IκBα levels were also reduced between minutes 10 and 30 in PIE cells prestimulated with *L. delbrueckii* TUA4408L or NPS while APS-treated cells showed no reduction in IκBα following poly(I:C) challenge. In addition, the three treatments significantly increased IκBα in PIE cells at minute 120 when compared with control cells (Figure 5). No significant changes were observed in the p-p38/p38 ratio in PIE cells after poly(I:C) challenge with the exception of NPS-treated cells that showed increased p-p38/p38 ratios at minutes 30 and 120 (Figure 5).

Poly(I:C) stimulation reduced TRAF3 at minute 30 (Figure 5). Similarly, diminished levels of TRAF3 were observed in APS–treated PIE at minute 30. NPS significantly increased TRAF3 at minutes 10, 30, and 120 while no changes were observed in TRAF3 levels in *L. delbrueckii*–treated PIE (Figure 5). In addition, enhanced p-IRF3/IRF3 ratio was observed in PIE cells 120 min after poly(I:C) challenge. However, in *L. delbrueckii*– and NPS–treated PIE cells, the p-IRF3/IRF3 ratio was significantly increased earlier at minute 10 while this increment was observed for APS treatment at minute 30 (Figure 5).

### *L. delbrueckii* TUA4408L and its EPSs modulate negative regulators of TLR signaling in PIE cells

**Figure 6:**
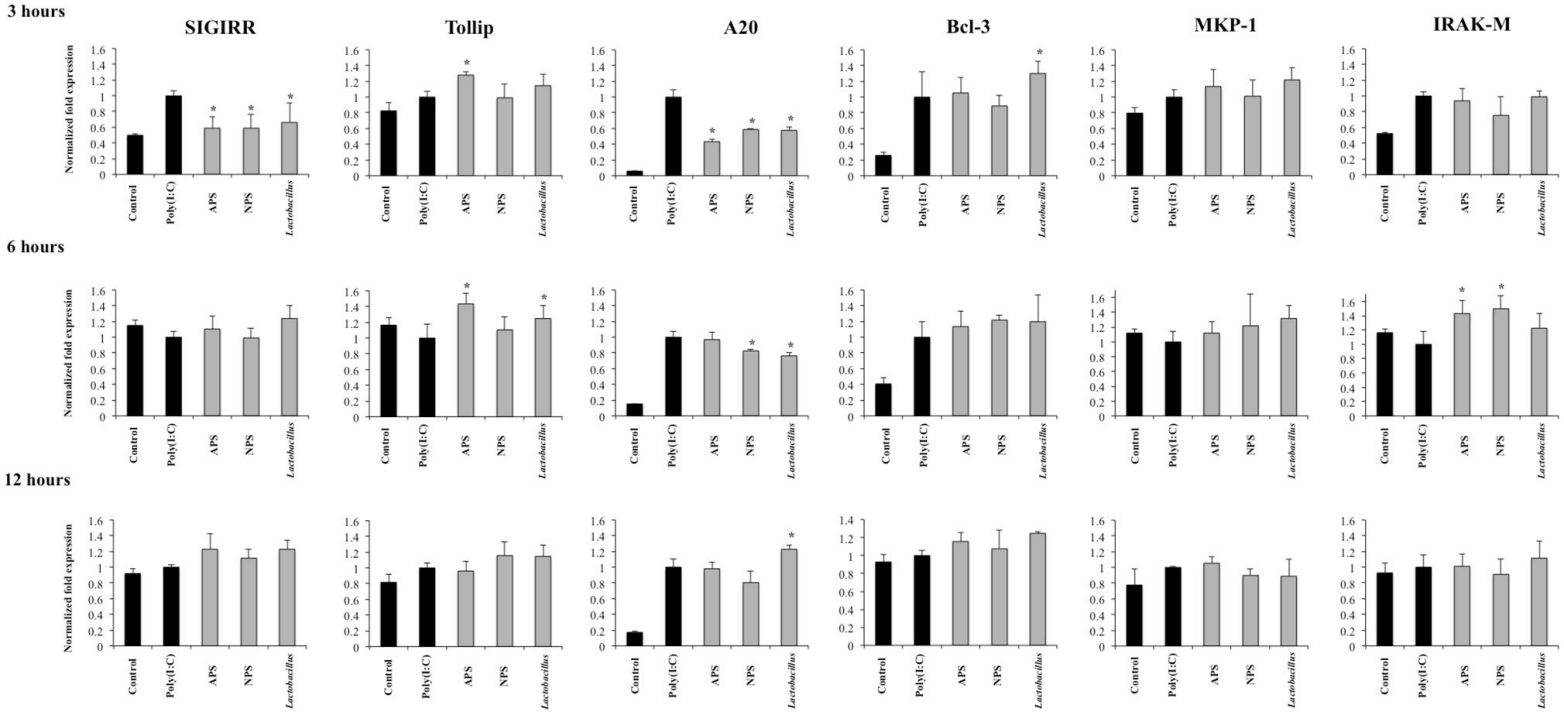
Effect of exopolysaccharides (EPS) from *Lactobacillus delbrueckii* subsp. *delbrueckii* TUA4408L on the expression of negative regulators of the Toll-like receptor (TLR) signaling in porcine intestinal epithelial (PIE) cells. EPS was fractionated into neutral (NSP) and acidic (APS) EPS by standard chromatographic methods. PIE cells were treated with lactobacilli, APS, NPS or medium for 48 h and then stimulated with the Toll-like receptor (TLR)-3 agonist poly(I:C). PIE cells without poly(I:C) stimulation were used as controls. The expression of single immunoglobulin IL-1-related receptor (SIGIRR), Toll interacting protein (Tollip), zinc finger protein A20 (A20), B-cell lymphoma 3-encoded protein (Bcl-3), mitogen-activated protein kinase-1 (MKP-1), and interleukin-1 receptor-associated kinase M (IRAK-M) was evaluated at 3, 6, and 12 h after poly(I:C) challenge. The results are expressed as mean ± SD and represent data from three independent experiments (*n* = 3 in each experiment). Asterisks indicate significant differences when compared to poly(I:C)-challenged control PIE cells (*P < 0.05).

Several intracellular and transmembrane proteins are known to modulate the activation of TLR3 acting as negative regulators. Considering the effects of *L. delbrueckii* TUA4408L and its EPSs on TLR3 signaling described above, we next aimed to evaluate whether these treatments were able to induce changes in the expression of several negative regulators of TLR3 in PIE cells after poly(I:C) challenge. The expression of the negative regulators: single immunoglobulin IL-1 related receptor (SIGIRR), toll interacting protein (Tollip), A20, B-cell lymphoma 3- encoded protein (Bcl-3), interleukin-1 receptor associated kinase M (IRAKM-1), and mitogen activated protein kinase phosphate 1 (MKP-1) were evaluated as shown in Figure 6.

Activation of TLR3 in PIE cells induced an early and strong up-regulation of A20 and Bcl-3 that was sustained during the studied period. In addition, increased expression of SIGIRR, Tollip, MKP-1, and IRAK-M was observed at hour 3 after poly(I:C) challenge. However, the expression of these four negative regulators returned to basal levels in hours 6 and 12 (Figure 6).

The three treatments *L. delbrueckii*, APS and NPS significantly reduced the expression of SIGIRR and A20 in poly(I:C)-challenged PIE cells early in hour 3 (Figure 6). Moreover, the expression of A20 remained reduced when compared to controls until hour 6 in *L. delbrueckii*- and NPS-treated PIE cells. Interestingly, A20 expression was significantly higher in *L. delbrueckii* TUA4408L-treated PIE cells at hour 12 when compared to the other experimental groups (Figure 6).

APS treatment was able to enhance the expression of Tollip and IRAK-M between hours 3 and 6 after poly(I:C) challenge while NPS increased the expression of IRAK-M at hour 6. In addition, *L. delbrueckii* TUA4408L-treated PIE cells showed an improved expression of Tollip and Bcl-3 at hours 6 and 3 after poly(I:C) challenge, respectively (Figure 6).

### Role of TLR2 and TLR4 on the immunomodulatory activities of *L. delbrueckii* TUA4408L and its EPSs

Previously, we demonstrated that the ability of *L. delbrueckii* TUA4408L and its EPSs to differentially modulate the immune response triggered by TLR4 activation is partially dependent on TLR2 and TLR4 (11). Then, we next aimed to find out whether the immunoregulatory effects observed in the context of TLR3 activation were also dependent on both TLRs. For this purpose, anti-TLR2 or anti-TLR4 blocking antibodies were used before the stimulation with *L. delbrueckii*, APS, or NPS, and the subsequent challenge with poly(I:C).

**Figure 7:**
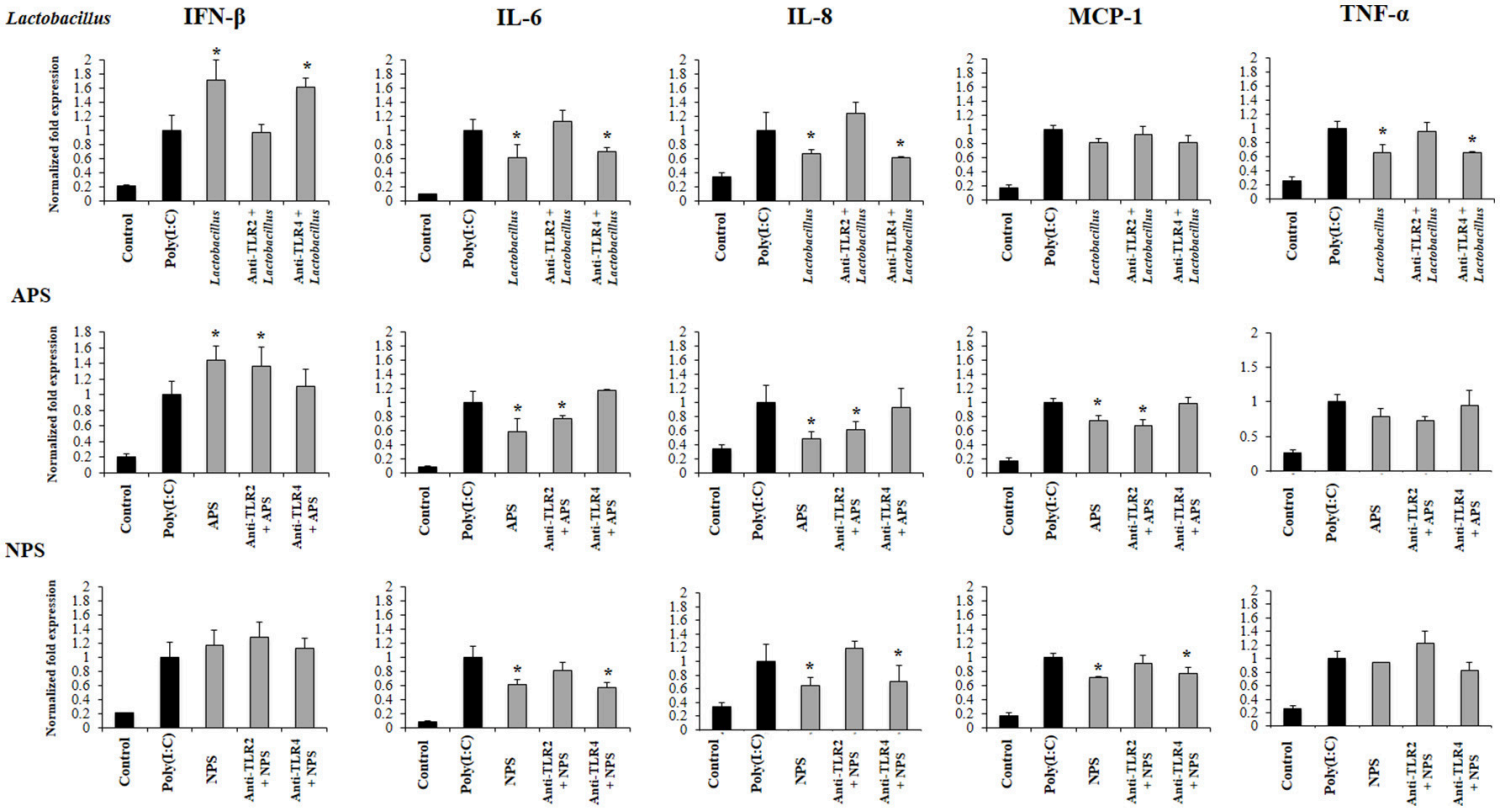
Role of Toll-like receptor (TLR)-2 and TLR4 in the immunomodulatory activity of exopolysaccharides (EPS) from *Lactobacillus delbrueckii* subsp. *delbrueckii* TUA4408L in porcine intestinal epithelial (PIE) cells. EPS was fractionated into neutral (NSP) and acidic (APS) EPS by standard chromatographic methods. PIE cells were incubated with anti-TLR2 or anti-TLR4 antibodies, treated with lactobacilli, APS or NPS for 48 h and, then stimulated with the TLR3 agonist poly(I:C). The expression of interferon β (IFN-β), interleukin 6 (IL-6), IL-8, monocyte chemoattractant protein-1 (MCP-1), and tumor necrosis factor (TNF-α) was evaluated 12 h after poly(I:C) challenge. The results are expressed as mean ± SD and represent data from three independent experiments (*n* = 3 in each experiment). Asterisks indicate significant differences when compared to poly(I:C)-challenged control PIE cells non treated with blocking antibodies (*P < 0.05).

The treatment of PIE cells with anti-TLR2 antibodies abolished the ability of *L. delbrueckii* to improve IFN-β and reduce the expression of IL-6, IL-8, and TNF-α after TLR3 activation, while anti-TLR4 antibodies did not induce significant changes in the immunomodulatory activity of the TUA4408L strain (Figure 7). Similarly, the treatment of PIE cells with anti-TLR2 antibodies abolished the ability of NPS to reduce the expression of IL-6, IL-8, and MCP-1 after TLR3 activation, while anti-TLR4 antibodies had no effect. On the contrary, anti-TLR2 antibodies did not induce modifications in the expression of inflammatory factors in APS-treated PIE cells. However, anti-TLR4 antibodies abolished the ability of APS to improve IFN-β and reduce inflammatory cytokines after TLR3 activation (Figure 7).

## Discussion

**Figure 8:**
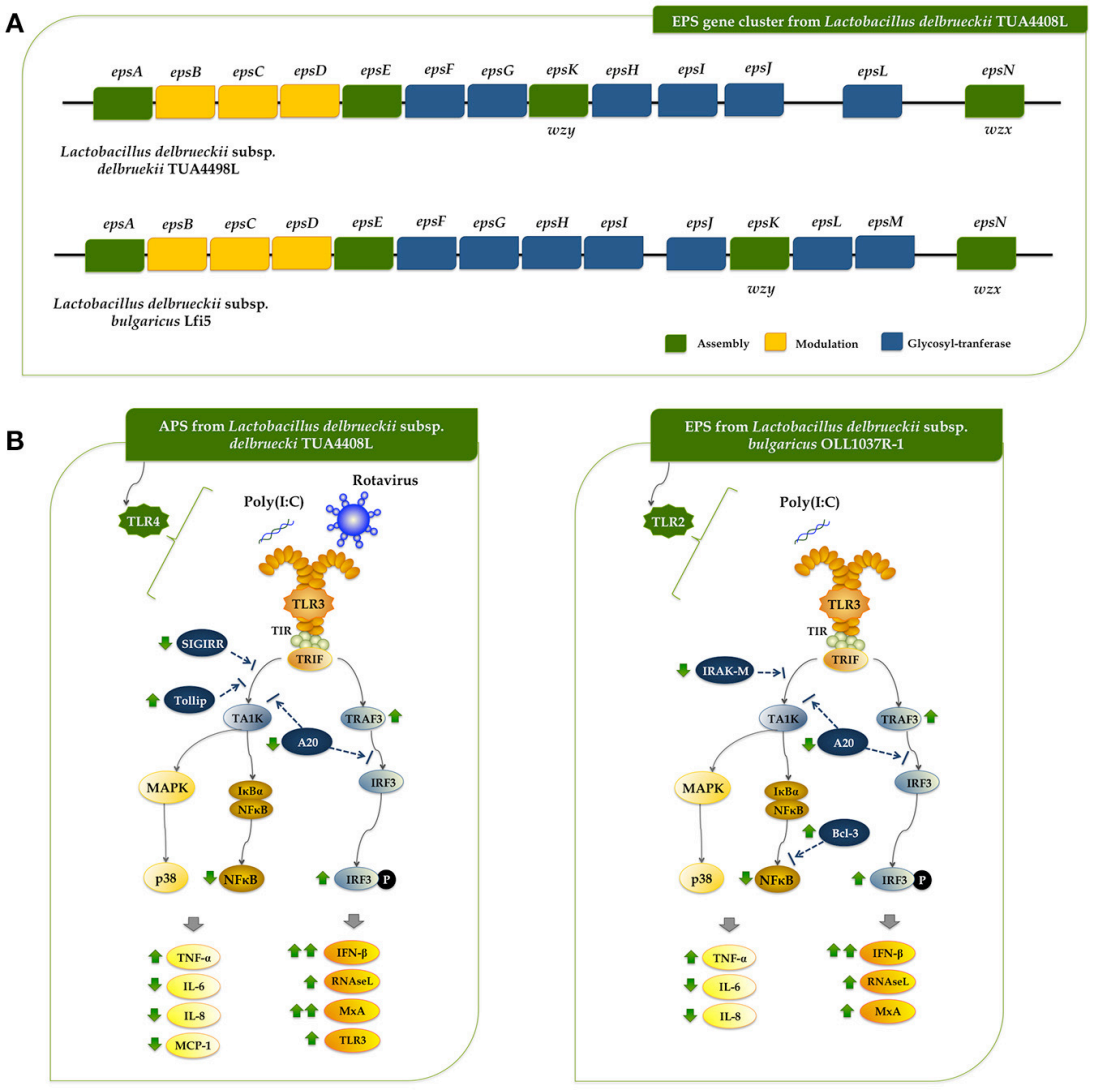
**(A)** Schematic genetic organization of the exopolysaccharides (EPS) gene cluster of *Lactobacillus delbrueckii* subsp. *delbrueckii* TUA4408L compared with the EPS gene cluster of *Lactobacillus delbrueckii* subsp. *bulgaricus* Lfi5. Gene functional grouping is marked with different colors. The nomenclature currently used for designating the genes in *eps* clusters encoding the Wzy-dependent pathway differs among organisms. For a generic LAB *eps* gene cluster, it was proposed to designate the five first conserved genes *epsABCDE*, the polymerase *wzy/epsK* and the flippase *wzx/epsN*. This nomenclature was used to designate those genes in the TUA4408L EPS cluster. In addition, the seven glycosiltransferases present in the TUA4408L EPS cluster were designated as *epsF* to *epsJ*, and *epsL* according to their location in the cluster. It should be noted that these genes are different from those with the same designation in the *L. bulgaricus* Lfi5 EPS cluster. **(B)** Proposed mechanisms for the antiviral activity of *L. delbrueckii* subsp. *delbrueckii* TUA4408L and its acidic extracellular polysaccharide (APS), and the EPS of *Lactobacillus delbrueckii* subsp. *bulgaricus* OLL1073R-1 in porcine intestinal epithelial (PIE) cells after stimulation with the Toll-like receptor (TLR)-3 agonist poly(I:C) or infection with rotavirus.

In this work, we reported and analyzed, for the first time, the complete genome of an industrial *L. delbrueckii* subsp. *delbrueckii* strain that has remarkable immunomodulatory properties (11). Genomic analysis revealed that the TUA4408L strain has an interesting metabolic potential relating to sugar assimilation and synthesis. This finding is in line with our results demonstrating the ability of the TUA44008L strain to growth and ferment several substrates such as pickles or soymilk, and to growth in several simple mediums containing only one source of sugars (unpublished data). In addition, we reported here the identification and genomic characterization of a chromosomally located EPS cluster from *L. delbrueckii* TUA4408L. The predicted gene products in the EPS cluster are homologous to proteins involved in the biosynthesis of other bacterial polysaccharides and the genetic organization was found to be similar to the EPS cluster from other species of lactobacilli (2, 32). In LAB, a typical EPS gene cluster consists of five highly conserved genes *epsA*-*epsE*, the polymerase *wzy*, the flippase *wzx*, and a variable number of glycosyltransferases and other polymer-modifying enzymes (2). This EPS gene cluster structure has been described and studied in *L. delbrueckii* subsp. *bulgaricus* Lfi5 (Figure 8A) (32). In *L. delbrueckii* TUA4408L genome, we found the genes encoding for the polysaccharide assembly machinery including those involved in the initiation of EPS biosynthesis (*epsE*), export (*wzx/epsN*), attachment (*epsA*), polymerization (*wzy/epsK*), as well as the phosphoregulatory module *epsBCD* (2). In addition, a variable region containing genes for seven putative glycosiltransferases was found in the TUA4408L EPS cluster (Figure 8A). It was also described that EPS gene clusters are highly diverse and their nucleotide sequences are among the most variable sequences in LAB genomes. In fact, insertion sequence elements flanking or within the operon are consistently present in the architecture of EPS gene clusters (36). We also have found this characteristic in the EPS cluster from *L. delbrueckii* TUA4408L.

A region containing seven genes for glycosyltransferases was found in *L. bulgaricus* Lfi5 EPS cluster (32), and the work suggested that all these putative glycosyltransferases were involved in the sequential biosynthesis of the EPS repeating unit. In fact, their number (seven) corresponded to the number of sugar residues present in the EPS repeating unit from Lfi5 strain (32). Preliminarily chemical evaluation of the EPS of *L. delbrueckii* TUA4408L indicated that the monosaccharaides glucose, mannose, galactose, arabinose and galacturonic acid are present in the EPS (unpublished data). Moreover, a branching glucose would be also present in the structure of the EPS repeating unit in the TUA4408L strain. Taking into consideration that glycosyltransferases constitute a large family of diverse proteins that have a wide range of enzymatic activities and functions, the prediction of the function of a putative glycosyltransferase by analyzing sequence homology is problematic because there are many examples of closely related sequences having different catalytic activity (37). Therefore, more detailed studies are necessary in order to characterize the glycosyltransferases *epsF* to *epsJ*, and *epsL* present in the *L. delbrueckii* TUA4408L EPS cluster as well as its chemical structure.

In addition to its genomic characterization, we have further advanced here in the study of the immunomodulatory properties of *L. delbrueckii* TUA4408L and its EPS. We have demonstrated that *L. delbrueckii* TUA4408L and its EPS are capable to regulate the innate immune response induced by the activation of TLR3 and improve the resistance of PIE cells against rotavirus infection.

Rotavirus is a naked double-stranded RNA (dsRNA) virus that infects mature intestinal epithelial cells. These cells sense rotavirus dsRNA through PRRs such as TLR3 and activate cellular signaling cascades to react to viral infection (33, 38). As a result of TLR3 activation, IFNs, IFN-regulated factors, cytokines, and chemokines are produced by infected cells in order to induce the recruitment and activation of immune cells and establish an antiviral state for virus clearance (33). Among the antiviral factors produced by infected cells, IFN-β is a key cytokine involved in the protection against rotavirus infection (39, 40), and therefore the improvement of this antiviral cytokine by intestinal epithelial cells have been used as a biomarker in the searching of beneficial microbes able to protect against rotavirus infection (reviewed in (33). In this regard, we have evaluated the suitability of PIE cells to be used as an efficient *in vitro* system for the search of beneficial microbes with antiviral capacities for humans and pigs. Our studies have shown that PIE cells have functional TLR3 which signal via IRF3 and NF-κB, inducing the up-regulation of IFN-β and the antiviral effectors MxA and RNaseL that are able to inhibiting viral replication and proliferation (41, 42). Furthermore, by studying IRF3 and NF-κB signaling pathway we have been able to find and characterize beneficial microbes with remarkable antiviral capacities including *L. casei* MEP221106 (43), *B. infantis* MCC12, *B. breve* MCC1274 (13), *L. rhamnosus* CRL1505, and *L. plantarum* CRL1506 (34, 35). In this work, by evaluating the innate antiviral immune response triggered by TLR3 activation and rotavirus infection in PIE cells we demonstrated that *L. delbrueckii* TUA4408L and its EPS are able to modulate IRF3 and NF-κB signaling pathways, improve IFN-β, MxA, and RNaseL expression, and significantly reduce rotavirus replication (Figure 8B).

In agreement with our previous studies (13, 34, 35, 43), we found that *L. delbrueckii* TUA4408L and its EPS differentially modulated the expression of several negative regulators of the TLR signaling pathway. In particular, a significant reduction in the expression of the zinc-finger protein A20 was observed in PIE cells treated with the APS, NPS or the TUA4408L strain. It was established that A20 is able to suppress IRF3 activation conducting to the reduction of the IFN-mediated immune response (44). Then, the reduction of A20 expression in EPS- or TUA4408L-treated PIE cells would be related the improved IRF3 activation and IFN-β, MxA, and RNaseL expression.

In addition to their ability to enhance antiviral effectors, microbes that improve protection against rotavirus infections have also the capacity to differentially modulate the expression of proinflammatory cytokines and chemokines (13, 34, 35, 43). The efficient regulation of inflammatory response induced viral attack is essential to achieve full protection against infection since unregulated inflammatory responses have been linked to cellular damage and severe mucosal injury in the gut during the course of rotavirus infection (33, 45). *L. delbrueckii* TUA4408L and its EPS have this characteristic since IL-6, IL-8, MCP-1, and TNF-α in PIE cells after TLR3 activation or rotavirus challenge were differentially expressed when compared to untreated PIE cells. The effect of the TUA4408L strain and its EPS on proinflammatory factors expression was probably coupled to their ability to differentially modulate the expression of the TLR negative regulators SIGIRR, Tollip, IRAK-M, and Bcl-3 (Figure 8B).

Besides epithelial cells, we have previously used porcine intestinal antigen presenting cells primary cultures to evaluate the effect of immunomodulatory probiotic strains (46) and such studies can be expanded to the analysis of the immunomodulatory capacities of *L. delbrueckii* subsp. *delbrueckii* and their EPS in antigen presenting cells in the future.

We were also interested in investigating whether TLR2 and TLR4 were involved in the antiviral activities of *L. delbrueckii* TUA4408L and its EPS since our previous studies evaluating their ability to influence immune responses in PIE cells triggered by TLR4 activation demonstrated that both PRRs were involved in their immunomodulatory effects (11). Our experiments using blocking antibodies, knockdown PIE cells, and calcium mobilization, demonstrated that the immunoregulatory capacities of *L. delbrueckii* TUA4408L EPS was dependent on TLR2 while APS exerted its immunomodulatory effect through TLR4 (11). In this work, we obtained similar results since the ability of the TUA4408L strain and its APS to modulate TLR3-triggered antiviral immune response in PIE cells was dependent on TLR2 and TLR4, respectively. It would be interesting to evaluate *in vivo* the role of TLR2 and TLR4 in the immunomodulatory effects of TUA4408L strain and its APS and also the role of TLR9 as this importantly relates to the production of immunosuppressive IL-10.

To the best of our knowledge, few studies have demonstrated the beneficial effects of EPS produced by lactobacilli in the improvement of innate antiviral immune response in general and in epithelial cells in particular. In a recent study, we evaluated the immunomodulatory activities of *L. delbrueckii* subsp. *bulgaricus* OLL1037R-1 and demonstrated that its EPS was able to induce a significant increase in the expression of IFN-α, IFN-β, MxA, and RNaseL in PIE cells after the stimulation of TLR3, in a TLR2 dependent manner (47). Interestingly, although EPS improved IFN-β, MxA, and RNaseL expression in PIE cells, this effect was not similar with purified APS or NPS from the OLL1073R-1 strain. In fact, our results indicated that the complete EPS molecule was necessary for obtaining the highest immunomodulatory/antiviral activity in PIE cells (47). On the other hand, the results presented here indicate that the beneficial effect induced by the TUA4408L strain can be reproduced completely by APS treatment, indicating that this EPS fraction is involved in the modulation of antiviral immunity. Then, our research works clearly demonstrated that the immunomodulatory properties of the EPS produced by lactobacilli are strain specific and therefore, the EPS of each potential probiotic strain should be studied in depth since it is not possible to make extrapolations even with strains of the same species. One question that remains open is how the APS from TUA4408L and the EPS from OLL1037R-1 acting through different receptors, TLR4 and TLR2 respectively, are able to induce almost identical effects on innate antiviral immunity (Figure 8B): improvement of IFN-β, TRAF3/IRF3, MxA, and RNaseL, reduction of A20 expression and NF-κB activation, and differential modulation of inflammatory cytokines and chemokines. The answer to this question is an important topic for future research.

The functional and genomic comparative studies of the effect of different EPS, APS, and NPS from *L. delbrueckii* subsp. *delbrueckii* strains with and without antiviral capabilities could be of great importance to deepen the knowledge of the molecular mechanisms related to its beneficial effects and to find those that are more efficient in the protection against viral infections. Moreover, the study and characterization of the EPS gene clusters of immunomodulatory lactobacilli, in particular of their variable regions, could provide bacterial biomarkers that allow an efficient screening in the search for EPS-producing strains with antiviral capabilities for its application in immunomodulatory functional foods and feeds. The genomic characterization of *L. delbrueckii* subsp. *delbrueckii* TUA4408L and the evaluation of the immunomodulatory/antiviral properties of its EPS reported here is an important advance in this line of research.

## Author contributions

HA, JV, and HaK designed the study and manuscript writing. PK, RK, YS, SE, and TM did the laboratory work related to antiviral immunity. HiK, and AM did the laboratory work related to rotavirus infection. LA, EH, LS, BG, and AS-B performed genomic analysis. PK, LA, HiK and JV did the statistical analysis. HA, YS, JV and HaK participated in the data analysis and integrative discussion. HT, YS, TM, and SE contributed to data analysis and interpretation. All the authors read and approved the manuscript.

### Conflict of interest statement

TM was employed by the company Marusan-Ai Co. Ltd. (Okazaki, Japan). The remaining authors declare that the research was conducted in the absence of any commercial or financial relationships that could be construed as a potential conflict of interest.

## References

[1] Ullrich M Bacterial Polysaccharides: Current Innovations and Future Trends. Horizon, TX: Scientific Press (2009).

[2] Zeidan AA Poulsen VK Janzen T Buldo P Derkx PMF Øregaard G. Polysaccharide production by lactic acid bacteria: from genes to industrial applications. FEMS Microbiol Rev. (2017) 41:S168–200. 10.1093/femsre/fux017 28830087

[3] Flemming HC Wingender J. The biofilm matrix. Nat Rev Microbiol. (2010) 8:623–33. 10.1038/nrmicro2415 20676145

[4] Caggianiello G Kleerebezem M Spano G. Exopolysaccharides produced by lactic acid bacteria: from health-promoting benefits to stress tolerance mechanisms. Appl Microbiol Biotechnol. (2016) 100:3877–86. 10.1007/s00253-016-7471-2 27020288

[5] Laiño J Villena J Kanmani P Kitazawa H. Immunoregulatory effects triggered by lactic acid bacteria exopolysaccharides: new insights into molecular interactions with host cells. Microorganisms (2016) 4:27. 10.3390/microorganisms4030027 27681921 PMC5039587

[6] Yasuda E Serata M Sako T. Suppressive effect on activation of macrophages by lactobacillus casei strain shirota genes determining the synthesis of cell wall-associated polysaccharides. Appl Environ Microbiol. (2008) 74:4746–55. 10.1128/AEM.00412-08 18552190 PMC2519339

[7] Bleau C Monges A Rashidan K Laverdure JP Lacroix M Van Calsteren MR. Intermediate chains of exopolysaccharides from Lactobacillus rhamnosus RW-9595M increase IL-10 production by macrophages. J Appl Microbiol. (2010) 108:666–75. 10.1111/j.1365-2672.2009.04450.x 19702865

[8] Balzaretti S Taverniti V Guglielmetti S Fiore W Minuzzo M Ngo HN. A novel rhamnose-rich hetero-exopolysaccharide isolated from *Lactobacillus paracasei* DG activates THP-1 human monocytic cells. Appl Environ Microbiol. (2017) 83:e02702–16. 10.1128/AEM.02702-16 27913418 PMC5244303

[9] Ciszek-Lenda M Nowak B Sróttek M Gamian A Marcinkiewicz J. Immunoregulatory potential of exopolysaccharide from *Lactobacillus rhamnosus* KL37. Effects on the production of inflammatory mediators by mouse macrophages. Int J Exp Pathol. (2011) 92:382–91. 10.1111/j.1365-2613.2011.00788.x 21950581 PMC3248074

[10] Moue M Tohno M Shimazu T Kido T Aso H Saito T. Toll-like receptor 4 and cytokine expression involved in functional immune response in an originally established porcine intestinal epitheliocyte cell line. Biochim Biophys Acta Gen Subj. (2008) 1780:134–44. 10.1016/j.bbagen.2007.11.006 18082146

[11] Wachi S Kanmani P Tomosada Y Kobayashi H Yuri T Egusa S. *Lactobacillus delbrueckii* TUA4408L and its extracellular polysaccharides attenuate enterotoxigenic *Escherichia coli*- induced inflammatory response in porcine intestinal epitheliocytes via Toll-like receptor-2 and 4. Mol Nutr Food Res. (2014) 58:2080–93. 10.1002/mnfr.201400218 24995380

[12] Kitazawa H Harata T Uemura J Saito T Kaneko T Itoh T. Phosphate group requirement for mitogenic activation of lymphocytes by an extracellular phosphopolysaccharide from Lactobacillus delbrueckii ssp. bulgaricus. Int J Food Microbiol. (1998) 40:169–75. 10.1016/S0168-1605(98)00030-0 9620124

[13] Ishizuka T Kanmani P Kobayashi H Miyazaki A Soma J Suda Y. Immunobiotic bifidobacteria strains modulate rotavirus immune response in porcine intestinal epitheliocytes via pattern recognition receptor signaling. PLoS ONE (2016) 11:e0152416. 10.1371/journal.pone.0152416 27023883 PMC4811565

[14] Chin CS Alexander DH Marks P Klammer AA Drake J Heiner C. Nonhybrid, finished microbial genome assemblies from long-read SMRT sequencing data. Nat Methods (2013) 10:563–9. 10.1038/nmeth.2474 23644548

[15] Seemann T. Prokka: rapid prokaryotic genome annotation. Bioinformatics (2014) 30:2068–9. 10.1093/bioinformatics/btu153 24642063

[16] Overbeek R Olson R Pusch GD Olsen GJ Davis JJ Disz T. The SEED and the Rapid Annotation of microbial genomes using Subsystems Technology (RAST). Nucleic Acids Res. (2014) 42:D206–14. 10.1093/nar/gkt1226 24293654 PMC3965101

[17] Grant JR Stothard P. The CGView Server: a comparative genomics tool for circular genomes. Nucleic Acids Res. (2008) 36:W181–4. 10.1093/nar/gkn179 18411202 PMC2447734

[18] Liu B Pop M. ARDB - Antibiotic resistance genes database. Nucleic Acids Res. (2009) 37: D443–7. 10.1093/nar/gkn656 18832362 PMC2686595

[19] Jia B Raphenya AR Alcock B Waglechner N Guo P Tsang KK. CARD 2017: expansion and model-centric curation of the comprehensive antibiotic resistance database. Nucleic Acids Res. (2017) 45:D566–73. 10.1093/nar/gkw1004 27789705 PMC5210516

[20] Bertelli C Laird MR Williams KP Lau BY Hoad G Winsor GL. IslandViewer 4: expanded prediction of genomic islands for larger-scale datasets. Nucleic Acids Res. (2017) 45:W30–5. 10.1093/nar/gkx343 28472413 PMC5570257

[21] Zhou Y Liang Y Lynch KH Dennis JJ Wishart DS. PHAST: A Fast Phage Search Tool. Nucleic Acids Res. (2011) 39:W347–52. 10.1093/nar/gkr485 21672955 PMC3125810

[22] Grissa I Vergnaud G Pourcel C. CRISPRcompar: a website to compare clustered regularly interspaced short palindromic repeats. Nucleic Acids Res. (2008) 36: W145–8. 10.1093/nar/gkn228 18442988 PMC2447796

[23] Tanigawa K Watanabe K. Multilocus sequence typing reveals a novel subspeciation of *Lactobacillus delbrueckii*. Microbiology (2011) 157:727–38. 10.1099/mic.0.043240-0 21178164

[24] Edgar RC. MUSCLE: multiple sequence alignment with high accuracy and high throughput. Nucleic Acids Res. (2004) 32:1792–7. 10.1093/nar/gkh340 15034147 PMC390337

[25] Kumar S Stecher G Tamura K. MEGA7: Molecular Evolutionary Genetics Analysis Version 7.0 for Bigger Datasets. Mol Biol Evol. (2016) 33:1870–4. 10.1093/molbev/msw054 27004904 PMC8210823

[26] Page AJ Cummins CA Hunt M Wong VK Reuter S Holden MTG. Roary: rapid large-scale prokaryote pan genome analysis. Bioinformatics (2015) 31:3691–3. 10.1093/bioinformatics/btv421 26198102 PMC4817141

[27] Bustin SA Benes V Garson JA Hellemans J Huggett J Kubista M. The MIQE guidelines: minimum information for publication of quantitative real-time PCR experiments. Clin Chem. (2009) 55:611–22. 10.1373/clinchem.2008.112797 19246619

[28] Nygard AB Jørgensen CB Cirera S Fredholm M. Selection of reference genes for gene expression studies in pig tissues using SYBR green qPCR. BMC Mol Biol. (2007) 8:67. 10.1186/1471-2199-8-67 17697375 PMC2000887

[29] Nilsen T Nes IF Holo H. Enterolysin A, a cell wall-degrading bacteriocin from *Enterococcus faecalis* LMG 2333. Appl Environ Microbiol. (2003) 69:2975–84. 10.1128/AEM.69.5.2975-2984.2003 12732574 PMC154489

[30] Suárez N Bonacina J Hebert E Saavedra L Genome mining and transcriptional analysis of bacteriocin genes in enterococcus faecium CRL1879. J Data Mining Genomics Proteomics (2015) 6:1 10.4172/2153-0602.1000180

[31] Makarova KS Wolf YI Alkhnbashi OS Costa F Shah SA Saunders SJ. An updated evolutionary classification of CRISPR–Cas systems. Nat Rev Microbiol. (2015) 13:722–36. 10.1038/nrmicro3569 26411297 PMC5426118

[32] Lamothe G Jolly L Mollet B Stingele F. Genetic and biochemical characterization of exopolysaccharide biosynthesis by *Lactobacillus delbrueckii subsp. bulgaricus*. Arch Microbiol. (2002) 178:218–28. 10.1007/s00203-002-0447-x 12189423

[33] Villena J Vizoso-Pinto MG Kitazawa H. Intestinal innate antiviral immunity and immunobiotics: beneficial effects against rotavirus infection. Front Immunol. (2016) 7:1–10. 10.3389/fimmu.2016.00563 27994593 PMC5136547

[34] Villena J Chiba E Vizoso-Pinto M Tomosada Y Takahashi T Ishizuka T. Immunobiotic *Lactobacillus rhamnosus* strains differentially modulate antiviral immune response in porcine intestinal epithelial and antigen presenting cells. BMC Microbiol. (2014) 14:126. 10.1186/1471-2180-14-126 24886142 PMC4035899

[35] Albarracin L Kobayashi H Iida H Sato N Nochi T Aso H. Transcriptomic analysis of the innate antiviral immune response in porcine intestinal epithelial cells: influence of immunobiotic *Lactobacilli*. Front Immunol. (2017) 8:57. 10.3389/fimmu.2017.00057 28210256 PMC5288346

[36] Cui Y Xu T Qu X Hu T Jiang X Zhao C. New insights into various production characteristics of *Streptococcus thermophilus* Strains. Int J Mol Sci. (2016) 17:1701. 10.3390/ijms17101701 27754312 PMC5085733

[37] Breton C Šnajdrová L Jeanneau C Koča J Imberty A. Structures and mechanisms of glycosyltransferases. Glycobiology (2006) 16:29R–37R. 10.1093/glycob/cwj016 16037492

[38] Frias AH Jones RM Fifadara NH Vijay-Kumar M Gewirtz AT. Rotavirus-induced IFN-β promotes anti-viral signaling and apoptosis that modulate viral replication in intestinal epithelial cells. Innate Immun. (2012) 18:294–306. 10.1177/1753425911401930 21733977

[39] Sen A Rothenberg ME Mukherjee G Feng N Kalisky T Nair N. Innate immune response to homologous rotavirus infection in the small intestinal villous epithelium at single-cell resolution. Proc Natl Acad Sci USA. (2012) 109:20667–72. 10.1073/pnas.1212188109 23188796 PMC3528539

[40] Meylan E Tschopp J. Toll-like receptors and RNA helicases: two parallel ways to trigger antiviral responses. Mol Cell (2006) 22:561–9. 10.1016/j.molcel.2006.05.012 16762830

[41] Liang SL Quirk D Zhou A. RNase L: Its biological roles and regulation. IUBMB Life (International Union Biochem Mol Biol Life) (2006) 58:508–14. 10.1080/15216540600838232 17002978

[42] Mitchell PS Patzina C Emerman M Haller O Malik HS Kochs G. Evolution-guided identification of antiviral specificity determinants in the broadly acting interferon-induced innate immunity factor MxA. Cell Host Microbe. (2012) 12:598–604. 10.1016/j.chom.2012.09.005 23084925 PMC3540999

[43] Hosoya S Villena J Shimazu T Tohno M Fujie H Chiba E. Immunobiotic lactic acid bacteria beneficially regulate immune response triggered by poly(I:C) in porcine intestinal epithelial cells. Vet Res. (2011) 42:111. 10.1186/1297-9716-42-111 22046952 PMC3220634

[44] Saitoh T Yamamoto M Miyagishi M Taira K Nakanishi M Fujita T. A20 is a negative regulator of IFN regulatory factor 3 signaling. J Immunol. (2005) 174:1507–12. 10.4049/jimmunol.174.3.1507 15661910

[45] Koyama S Ishii KJ Coban C Akira S. Innate immune response to viral infection. Cytokine (2008) 43:336–41. 10.1016/j.cyto.2008.07.009 18694646

[46] Tsukida K Takahashi T Iida H Kanmani P Suda Y Nochi T Immunoregulatory effects triggered by immunobiotic *Lactobacillus jensenii* TL2937 involve efficient phagocytosis in porcine dendritic cells. BMC Immunol. (2016) 17:21 10.1186/s12865-016-0160-1 27342653 PMC4921007

[47] Kanmani P Albarracin L Kobayashi H Iida H Komatsu R Humayun Kober AKM. Exopolysaccharides from *Lactobacillus delbrueckii* OLL1073R-1 modulate innate antiviral immune response in porcine intestinal epithelial cells. Mol Immunol. (2018) 93:253–65. 10.1016/j.molimm.2017.07.009 28800975

