# Supplementary material for: Genomic Characterization of Lactobacillus delbrueckii TUA4408L and Evaluation of the Antiviral Activities of its Extracellular Polysaccharides in Porcine Intestinal Epithelial Cells
Source: Front Immunol. 2018 Sep 24;9:2178. doi: 10.3389/fimmu.2018.02178 (PMC6165883; doi:10.3389/fimmu.2018.02178)
Supplement: Supplementary file 1 [file Data_Sheet_1.doc]

**Supplemental Table 1**. List of unique genes found in *L. delbrueckii* subsp. *delbrueckii* TUA4408L genome after pangenomic analysis and comparison with *L. delbrueckii* subsp. *delbrueckii* strains DSM 20074, NBRC 2302, KCTC 13731, and KACC 13439. *L. delbrueckii* TUA4408L has 338 unique genes including 136 proteins with known functions and 202 hypothetical proteins (not shown). Unique genes related to sugar metabolism are shown in the table.

| ***Gene*** | **Synonyms** | **Gene annotation** | **Locus_tag** | **Function related to sugar metabolism** |
| --- | --- | --- | --- | --- |
| *group_2427* | *gmuC_1* | PTS system oligo-beta-mannoside-specific EIIC component | TUA4408L_00136 | The phosphoenolpyruvate-dependent sugar phosphotransferase system (PTS), a major carbohydrate active -transport system, catalyzes the phosphorylation of incoming sugar substrates concomitant with their translocation across the cell membrane. The enzyme II GmuABC PTS system is involved in the transport of oligo-glucomannans such as cellobiose or mannobiose. |
| *licA* |  | Lichenan-specific phosphotransferase enzyme IIA component | TUA4408L_00137 | PTS protein form the system is involved in lichenan transport. |
| *group_2429* | *bglA_1* | Aryl-phospho-beta-D-glucosidase BglA | TUA4408L_00139 | Catalyzes the hydrolysis of aryl-phospho-beta-D-glucosides such as 4-methylumbelliferyl-phospho-beta-D-glucopyranoside (MUG-P), phosphoarbutin and phosphosalicin. Plays a major role in the utilization of arbutin or salicin as the sole carbon source. |
| *levS* |  | Levansucrase | TUA4408L_00388 | This enzyme belongs to the family of glycosyltransferases (hexosyltransferases). It is also called sucrose 6-fructosyltransferase or beta-2,6-fructosyltransferase. This enzyme participates in starch and sucrose metabolism and two-component system. |
| *glgA* |  | Glycogen synthase | TUA4408L_00653 | Synthesizes alpha-1,4-glucan chains using ADP-glucose. |
| *glgD* |  | Glycogen biosynthesis protein GlgD | TUA4408L_00654 | Enzyme required for the synthesis of glycogen. |
| *glgC* |  | Glucose-1-phosphate adenylyltransferase | TUA4408L_00655 | This protein plays a role in synthesis of starch. It catalyzes the synthesis of the activated glycosyl donor, ADP-glucose from Glc-1-P and ATP. |
| *glgB* |  | 1,4-alpha-glucan branching enzyme GlgB | TUA4408L_00656 | Essential enzyme that catalyzes the formation of the alpha-1,6-glucosidic linkages in glucan chains by scission of a 1,4-alpha-linked oligosaccharide from growing alpha-1,4-glucan chains and the subsequent attachment of the oligosaccharide to the alpha-1,6 position. Is involved in the biosynthesis of both glycogen and capsular alpha-D-glucan. |
| *group_853* | *malP_2* | PTS system maltose-specific EIICB component | TUA4408L_00689 | This is a major carbohydrate active transport system, catalyzes the phosphorylation of incoming sugar substrates concomitantly with their translocation across the cell membrane. This system is involved in maltose transport. |
| *epsF_1* |  | Putative glycosyltransferase EpsF | TUA4408L_00794 | This is involved in the production of the exopolysaccharide (EPS) component of the extracellular matrix during biofilm formation. EPS is responsible for the adhesion of chains of cells into bundles. Required for biofilm maintenance. |
| *group_1847* | *glf* | UDP-galactopyranose mutase | TUA4408L_00796 | Catalyzes the interconversion through a 2-keto intermediate of uridine diphosphogalactopyranose (UDP-GalP) into uridine diphosphogalactofuranose (UDP-GalF). It is involved in the biosynthesis of galactofuranose glycoconjugates. |
| *group_2472* | *pglC* | Undecaprenyl phosphate N,N'-diacetylbacillosamine 1-phosphate transferase | TUA4408L_00798 | Glycosyl-1-phosphate transferase that mediates the first step in the biosynthesis of the undecaprenyl-linked heptasaccharide donor in the N-linked protein glycosylation pathway. Catalyzes the linking of uridine 5'-diphosphobacillosamine (UDP-Bac) to undecaprenyl phosphate to create the first membrane-associated intermediate undecaprenylpyrophosphate-linked Bac (Und-PP-Bac). |
| *group_2473* | *inuJ* | Inulosucrase | TUA4408L_00801 | Fructosyltransferase that catalyzes the polymerization of the fructose moiety of sucrose to produce inulin polymer and inulin oligosaccharides such as 1-kestose and nystose. |
| *licC* |  | Lichenan permease IIC component | TUA4408L_00837 | This is a major carbohydrate active -transport system, catalyzes the phosphorylation of incoming sugar substrates concomitant with their translocation across the cell membrane. This system is involved in lichenan transport |
| *celA* |  | PTS system cellobiose-specific EIIB component | TUA4408L_00838 | Phosphotransferase system (sugar PTS) involved in cellobiose transport. |
| *celD* |  | PTS system cellobiose-specific EIIA component | TUA4408L_00839 | Phosphotransferase system (sugar PTS) involved in cellobiose transport. |
| *group_2493* | *rfbX_2* | Putative O-antigen transporter | TUA4408L_00847 | Capsular biosynthesis protein |
| *group_2497* | *epsJ_1* | putative glycosyltransferase EpsJ | TUA4408L_00858 | It is involved in the production of the exopolysaccharide (EPS) component of the extracellular matrix during biofilm formation. |
| *group_2498* | *epsJ_2* | putative glycosyltransferase EpsJ | TUA4408L_00859 | It is involved in the production of the exopolysaccharide (EPS) component of the extracellular matrix during biofilm formation. |
| *group_2499* | *epsG* | Transmembrane protein EpsG | TUA4408L_00860 | It is involved in the production of the exopolysaccharide (EPS) component of the extracellular matrix during biofilm formation. |
| *epsF_2* |  | Putative glycosyltransferase EpsF | TUA4408L_00862 | It is involved in the production of the exopolysaccharide (EPS) component of the extracellular matrix during biofilm formation. |
| *glgM* |  | Alpha-maltose-1-phosphate synthase | TUA4408L_00863 | Involved in the biosynthesis of the maltose-1-phosphate (M1P) building block required for alpha-glucan production by the key enzyme GlgE. lso able to catalyze the elongation of the non-reducing ends of glycogen, maltodextrin and maltoheptaose using ADP-glucose as sugar donor. |
| *group_2504* | *cap8A* | Capsular polysaccharide type 8 biosynthesis protein cap8A | TUA4408L_00867 | Required for the biosynthesis of type 8 capsular polysaccharide (Cap8/CP8). Might act as the chain-length regulator. |
| *crr_3* |  | PTS system glucose-specific EIIA component | TUA4408L_00923 | The enzyme II complex composed of PtsG and Crr is involved in glucose transport. Also functions as a chemoreceptor monitoring the environment for changes in sugar concentration and an effector modulating the activity of the transcriptional repressor Mlc. |
| *ttdT* |  | L-tartrate/succinate antiporter | TUA4408L_00974 | Catalyzes the uptake of tartrate in exchange for intracellular succinate. Essential for anaerobic L-tartrate fermentation. |
| *ndvA* |  | Beta-(1-->2)glucan export ATP-binding/permease protein NdvA | TUA4408L_01047 | Involved in beta-(1-->2)glucan export. Transmembrane domains (TMD) form a pore in the inner membrane and the ATP-binding domain (NBD) is responsible for energy generation. |
| *mngR* |  | Mannosyl-D-glycerate transport/metabolism system repressor MngR | TUA4408L_01104 | This protein represses mngA and mngB, genes involved in in mannosyl-D-glycerate transport. It also regulates its own expression. |
| *chbB* |  | PTS system N,N'-diacetylchitobiose-specific EIIB component | TUA4408L_01105 | The enzyme II ChbABC PTS system is involved in the transport of the chitin disaccharide N,N'-diacetylchitobiose (GlcNAc2) |
| *group_2528* | *gmuC_2* | PTS system oligo-beta-mannoside-specific EIIC component | TUA4408L_01106 | The enzyme II GmuABC PTS system is involved in the transport of oligo-glucomannans such as cellobiose or mannobiose. |
| *lacF_2* |  | PTS system lactose-specific EIIA component | TUA4408L_01107 | The enzyme II LacEF PTS system is involved in lactose transport, but can also use galactose, isopropyl beta-thio-galactopyranoside and thiometyl beta-D-galactopyranoside (TMG) as substrates. |
| *bglK_2* |  | Beta-glucoside kinase | TUA4408L_01109 | Catalyzes the ATP-dependent phosphorylation of a wide variety of beta-D-glucosides, to produce 6-phospho-beta-D-glucosides including cellobiose-6'-P, gentiobiose-6'-P, cellobiitol-6-P, salicin-6-P, and arbutin-6-P. May have a dual role of kinase and transcriptional regulator of the cellobiose-PTS operon. |
| *sugC_1* |  | Trehalose import ATP-binding protein SugC | TUA4408L_01143 | Part of the ABC transporter complex LpqY-SugA-SugB-SugC, which is highly specific for uptake of trehalose. |
| *lacF_3* |  | Lactose transport system permease protein LacF | TUA4408L_01145 | The enzyme II LacEF PTS system is involved in lactose transport. |
| *araQ* |  | L-arabinose transport system permease protein AraQ | TUA4408L_01146 | Part of the binding-protein-dependent transport system for L-arabinose. Probably responsible for the translocation of the substrate across the membrane. |
| *msmE* |  | Multiple sugar-binding protein | TUA4408L_01148 | Transmembrane transporter activity |
| *xpkA_1* |  | Xylulose-5-phosphate phosphoketolase | TUA4408L_01540 | Phosphoketolase using both fructose 6-phosphate and xylulose 5-phosphate as substrate. |
| *rbsB* |  | Ribose import binding protein RbsB | TUA4408L_01998 | Part of the ABC transporter complex RbsABC involved in ribose import. Binds ribose. |
| *manX_2* |  | PTS system mannose-specific EIIAB component | TUA4408L_01999 | The enzyme II ManXYZ PTS system is involved in mannose transport. |
| *sorB* |  | PTS system sorbose-specific EIIB component | TUA4408L_02000 | The enzyme II SorABCD PTS system is involved in L-sorbose transport. |
| *sorA_2* |  | PTS system sorbose-specific EIIC component | TUA4408L_02001 | The enzyme II SorABCD PTS system is involved in L-sorbose transport. |
| *manZ_2* |  | PTS system mannose-specific EIID component | TUA4408L_02002 | The enzyme II ManXYZ PTS system is involved in mannose transport. |
