# Supplementary material for: Genomic Characterization of Lactobacillus delbrueckii TUA4408L and Evaluation of the Antiviral Activities of its Extracellular Polysaccharides in Porcine Intestinal Epithelial Cells
Source: Front Immunol. 2018 Sep 24;9:2178. doi: 10.3389/fimmu.2018.02178 (PMC6165883; doi:10.3389/fimmu.2018.02178)
Supplement: Supplementary file 2 [file Data_Sheet_2.doc]

**Supplemental Table 2**. List of conserved genes in the exopolysaccharides (EPS) cluster of *L. delbrueckii* subsp. *delbrueckii* TUA4408L compared with the EPS cluster of *L. delbrueckii* subsp. *bulgaricus* Lfi5.

| **Gene** | **Function** | **TUA4408L compared with Lfi5** | **References** |
| --- | --- | --- | --- |
| *epsA* | The gene product be involved in the regulation of EPS synthesis in a positive manner. It is homologous to *CpsIaA* from *S. agalactiae*, which has been shown to be an activator of capsule gene transcription.  Transcriptional regulation of the eps operon in lactic acid bacteria has long been attributed to *EpsA*.  Data from *L. johnsonii* FI9785 suggested that EpsA is a positive regulator of the *eps* operon | Query: 100%  Identity: 94% | (14,15)  (16,17)  (18) |
| *epsB* | The gene product is involved in chain-length determination of EPS. EpsB is homologue of CpsC from *S. pneumoniae* which have been shown to exhibit a tyrosine kinase activity. | Query: 100%  Identity: 93% | (14,19) |
| *epsC* | The gene product is involved in chain-length determination of EPS. EpsC is homologues of CpsD from *S. pneumoniae* which have been shown to exhibit a tyrosine kinase activity.  It belongs to the *EpsBCD* phosphoregulatory system. EpsC is a transmembrane activation protein that is required for the autophosphorylation of the tyrosine cluster of the cytoplasmic protein EpsD. | Query: 98%  Identity: 96% | (14,19)  (20,21) |
| *epsD* | The gene product is involved in chain-length determination of EPS.  It belongs to the *EpsBCD* phosphoregulatory system. Together with EpsC proteins constitute a functional autophosphorylating BY kinase, the activity of which is modulated via interaction with the phosphotyrosine phosphatase EpsB. | Query: 98%  Identity: 97% | (14)  (20,21) |
| *epsE* | The gene product is a phosphor-glucosyltransferase initiating the biosynthesis of EPS.  It belongs to the *EpsBCD* phosphoregulatory system. | Query: 100%  Identity: 92% | (14) |
| *wzy* (*epsK*) | The gene product is homologous to Wzy from *E. coli*, known to be the polymerase of O-antigen units. It possesses a large number of predicted transmembrane segments.  The Wzy polymerases are membrane-bound proteins putatively harboring 10 to 14 transmembrane segments. The role of theWzy polymerase is to add a single repeating unit via generation of a new glycosidic bond to the reducing terminus of a polysaccharide composed of multiple copies of the repeating unit. | Query: 100%  Identity: 92% | (14,22)  (23) |
| *wzx* (*epsN*) | The gene product is involved in EPS export because and has homology to Wzx from *E. coli*, widely referred to as “flippase”, which translocates undecaprenyl-linked O-antigen units across the membrane.  The Wzx flippases are membrane-bound proteins often characterized by the presence of 12 transmembrane segments, which are classified as part of the polysaccharide transporter family. Wzx flippases recognize the UndP-P-repeating unit and flip it across the cytoplasmic membrane. | Query: 100%  Identity: 81% | (14,24)  (23) |
