# Supplementary material for: Genomic Characterization of Lactobacillus delbrueckii TUA4408L and Evaluation of the Antiviral Activities of its Extracellular Polysaccharides in Porcine Intestinal Epithelial Cells
Source: Front Immunol. 2018 Sep 24;9:2178. doi: 10.3389/fimmu.2018.02178 (PMC6165883; doi:10.3389/fimmu.2018.02178)
Supplement: Supplementary file 3 [file Data_Sheet_3.doc]

**Supplemental Table 3**. List of genes codifying for glycosyltransferases in the exopolysaccharides (EPS) cluster of *L. delbrueckii* subsp. *delbrueckii* TUA4408L.

| **Gene**  **(Prokka)** | **Gene designation in this work** | **Classification and function**  **(Blast – Uniprot)** | **Aminoacid sequence** |
| --- | --- | --- | --- |
| *Peg824*  *rafB* | *epsF* | Glycosyltransferase GTB type superfamily  (Glycosyl transferases group 1 = Glyco_trans_1)  Proteins containign this domain transfer UDP, ADP, GDP or CMP linked sugars to a variety of substrates. The bacterial enzymes are involved in various biosynthetic processes that include exopolysaccharide biosynthesis.  UNIPROT: 67.1% of identity with A0A1A7SPW8 (DTPHA_602344 gene) from *Enterococcus faecium* strain Isolate 22. | MKNHLGKIKSGPLRIAMLGHKEIHSRQGGIEVVVEELATRMVKMGHQVTAFNRSSHNVAGREFDVKRRNTYKGIRLKYVPTLDKKGLAAVTSSFFGAVAAAFGRYDIVHFHAEGPSSMLWIPKMFGKRCIVTIHGLDHQRAKWGKFASAYIMMGEKCAVKMADEIIVLSQDMKDYFSKEYGRDTVFIPNGVNRPNMAGDSLIQEEYGLKKDDYILFLGRLVPEKGLSYLVEAFKKTKTDKKLVIAGGNSDTQDFTEQLKEQAKDDNRIIFTGFVQGEMLETLYSNSYIYVLPSDLEGMPLSLLEAMSYGNCCLVSDIDECASVVEDKAVLFKKSDIVDLQNKLQNLCNNPALVKKYKDEASDFICNKYNWEKIVDDTLELYRGIYHD |
| *Peg823*  *capH* | *epsG* | Glycosyltransferase GTB type superfamily  (GT1_CapH_like related to GT1 family of glycosyltransferases)  In *Staphylococcus aureus* has been shown to be required for the biosynthesis of the type 1 capsular polysaccharide (CP1).  UNIPROT: 68.3% of identity with A0A173YSV9 the mannosylfructose-phosphate synthase from *Turicibacter sanguinis* and 64.5% of identity with A0A0D5MJN7 (HUO_10095 gene) from *Lactobacillus helveticus* strain KLDS1.8701.Similarities with mannosylfructose-phosphate synthase from several other bacterial species. | MIRVLQVVNDMHRAGLETMLMNYYRNIDRDQIQFDFLTHRPYKSDYDDEIISLGGKVYYAPRLYPQNYPAYFKWMKQFFAEHPEYHIVHSHIDSMSYLPLRAAKKAGIPHRIAHSHNTSIDKDFKYPLKQLFRFELPHVANHYLACGQDAGKFMYGNRHFIVIPNAVEADKFYFNERVRKMKRSALGLVDDQFVVGHVGRLSYQKNHKFILQIFNALSKKDSKAVLLLVGTGEKEEEIRSQIKEVGLENKVMMLGNRGDVSELYQAMDVFILPSLFEGIPVVGVESQFAGLPVLFSDKVPTEVKFSSKCQFLSLDATPTEWANKILKSKSTKRTGNYEDLKDSPYDIKNSHDILENYYLNFDGVAND |
| *Peg821* | *epsH* | Glyco_tranf_2_3 superfamily  (Glycoside transferase family 2)  UNIPROT: 32.2% of identity with an uncharacterized protein from *Lactobacillus gallinarum* DSM 10532 = JCM 2011 and 32.1% of a glycosyltrasferase from *Lactobacillus crispatus* EM-LC1. 34.9% of identity with *Lactobacillus saerimneri 30a.* 36.5% *Lactobacillus nodensis* DSM 19682 = JCM 14932 = NBRC 107160. | METKPNISFIIPVFNEELAKLKRCIESITSNTKNVDYEILLVDDGSLSQNSEKYKKIARELRVEYIHQLNGGVSSARNLGIDHAHGNYIGFIDSDDELGTIDYRSAVKTNADFLIFDIDVLNGKQQHFIRQNIPKKEFADTQKMLLDSVTSFNISNPFGKLYKTDFIKSHGIKFDTTLKQGEDYDFVVQVLYQKPTVYYIDQVEYLYHQQQSSSIKRLRSFPSILVDNYLHEYELKARIVSDFAPNNTDLMQRIDSDLVKNMFSIMAEALGLKTKEAYEARKKIISSMSKIEDPSKLSKMSKIKFRAMEQNNYLLLSILKVMRAFYIKLRY |
| *Peg820* | *epsI* | Glyco_tranf_2_3 superfamily  (Glycoside transferase family 2)  UNIPROT: 38.2% of glycosyltransferase from *Lactobacillus mali* KCTC 3596 = DSM 20444, 36.7% of identity with Beta-1-4 galactosyltransferase from *Lactobacillus buchneri* CD034. | MFNFKKVAKFCYYKIKVAQYIASNFRGSVNNELMSVDEARKTLSSVRPQPKGKVTRDWEIISDESVDLSIIVPVYNVEDTLKQCLDSCFSQKTKYSFEVIAINDGSPDNSGAILAEYKDERLKVITQENKGLSGARNTGLLARHGKYVLFLDSDDYLFPGAIEVLLNAALKSHADIVEGSYLNLRSGKLLSGFKHPTGCHKIQSYHDLYGFPWGKVYTSRIFNELEFPQDYIFEDTVVGMLWIPISKSIYTLGDNVLVYRTNPKGISATVGNEDRSIDSYWITELLVEEQSKRDLLNQENFSFFLDQLAINYFRIRQMDKKIKLAEFVLSRALLNVYLESSSVQVERLSGKKIKLVKHMLAGDYRKTIDLLAAWEGMK |
| *Peg819* | *epsJ* | Caps-Synth superfamily  Capsular polysaccharide synthesis protein; This family consists of several capsular polysaccharide proteins.  UNIPROT: 86.1% of identity with an uncharacterized protein from *Lactobacillus delbrueckii* subsp. *bulgaricus* CRL871 and around 30% of identity with Alpha-1- 2- galactosyltransferase EpsI from several lactobacilli strains. | MKQLIVDKFKYNYLKKYTQILGKFDKLYPLPGAYKRLHEKTLKCLKQDLDVVDGYFDEIGFSKMSSEDIFSEGKIWIMWWQGYNEAPKLVKKNIDTLTNLFGEEKVQVITKENYKQFVTISTSILKRVEFGQLGITGFSDVIRYNLLAAHGGIWMDSTVVASDLAYDFIKEREGQNFITLKEGQNNYHFISNAKWATWFIGGKKGYPLFQYVAKFYDLLLISQGFTTQAIRLTDFLPAVFLLCMTFQKCEDFSFSSGTFINSF |
| *Peg817* | *epsL* | Branch superfamily  (Core-2/I-Branching enzyme)  This is a family of two different beta-1,6-N-acetylglucosaminyltransferase enzymes, I-branching enzyme and core-2 branching enzyme.  UNIPROT: 48.6% of a putative glycosyltransferase from *Lactobacillus salivarius* NIAS840 and 47.2% of identity with the acetylglucosaminyltransferase of *Lactobacillus gallinarum* DSM 10532 = JCM 2011 | MKKIDFNLTVLYLGYTMNQSINQPTNQAIMVIGHGDSAPVLQETINVLDNEKIDFFIHWDKKNLQPKLYSKKSKIFFVDPIEVRWGTDTQIKAELLLMQAVQRHGGYTMAHLISAADMPLMDANYFLNYFDGRKSYLGFSQTPNNIGEDIVQRIKYYWPDRDLKGHKLYIRLFIAKNKLLGTDRLKDHTLQLYKGANWFSINTTYFDKVIMADKTPFFFCADELFMQTILPELNWQDNKVDDNTQAARYVDWGRGTPYTFKIDDVMELERVKNTKYAFARKVTDPSVVKAVFV |
